# miRNA cargo in circulating vesicles from neurons is altered in individuals with schizophrenia and associated with severe disease

**DOI:** 10.1126/sciadv.adi4386

**Published:** 2023-11-29

**Authors:** Michelle M. Barnett, William R. Reay, Michael P. Geaghan, Dylan J. Kiltschewskij, Melissa J. Green, Judith Weidenhofer, Stephen J. Glatt, Murray J. Cairns

**Affiliations:** ^1^ School of Biomedical Sciences and Pharmacy, Faculty of Health and Medicine, The University of Newcastle, Callaghan, NSW 2308, Australia.; ^2^ Precision Medicine Research Program, Hunter Medical Research Institute, Newcastle, NSW 2305, Australia.; ^3^ Kinghorn Centre for Clinical Genomics, Garvan Medical Research Institute, Darlinghurst, NSW 2010, Australia.; ^4^ Discipline of Psychiatry and Mental Health, School of Clinical Medicine, University of New South Wales, Sydney, NSW 2052, Australia.; ^5^ Neuroscience Research Australia, Sydney, NSW, Australia.; ^6^ Psychiatric Genetic Epidemiology and Neurobiology Laboratory (PsychGENe lab), Department of Psychiatry and Behavioral Sciences, SUNY Upstate Medical University, Syracuse, NY, USA.

## Abstract

While RNA expression appears to be altered in several brain disorders, the constraints of postmortem analysis make it impractical for well-powered population studies and biomarker development. Given that the unique molecular composition of neurons are reflected in their extracellular vesicles (EVs), we hypothesized that the fractionation of neuron derived EVs provides an opportunity to specifically profile their encapsulated contents noninvasively from blood. To investigate this hypothesis, we determined miRNA expression in microtubule associated protein 1B (MAP1B)–enriched serum EVs derived from neurons from a large cohort of individuals with schizophrenia and nonpsychiatric comparison participants. We observed dysregulation of miRNA in schizophrenia subjects, in particular those with treatment-resistance and severe cognitive deficits. These data support the hypothesis that schizophrenia is associated with alterations in posttranscriptional regulation of synaptic gene expression and provides an example of the potential utility of tissue-specific EV analysis in brain disorders.

## Teaser

Analysis of neuronal miRNA from serum vesicles provides a molecular signature of neuropathology in schizophrenia.

## INTRODUCTION

Biomarkers for brain disorders are desperately needed to support objective diagnosis, early intervention, and precision medicine. The sampling of living brain tissue for biopsy, however, is not feasible given the technical and ethical challenges, which has led to the application of peripheral or circulating tissue, such as blood and serum as a proxy for changes associated with neurological and psychiatric pathology. Despite their accessibility, circulating cellular and extracellular biomolecules are confounded by their composition and proximity to a vast array of tissues with divergent functions. Where circulating biomolecules are tagged by their tissue of origin, such as the brain, there is potential to establish a more direct relationship with the pathology and generate biomarkers with much greater signal to noise ratio.

Extracellular vesicles (EVs) are abundant in the circulation and extracellular fluid, and encapsulate a large array of proteins, nucleic acids and metabolites that mirror the local environment at their tissue of origin. Their encapsulating lipid bilayer is also composed of plasma membrane with tissue-specific ligands, or antigens, that provide an opportunity to discriminate their parental cell type. EVs are also highly enriched with microRNAs (miRNAs) (*1*) that have been shown to influence the abundance and function of mRNA in the brain (*2*–*4*). Alterations in brain miRNA impair synaptic plasticity (*5*, *6*) and are associated with many human diseases, including psychiatric disorders (*7*, *8*). While miRNAs appear to be dysregulated in postmortem brain tissue derived from schizophrenia (SZ) cases, existing studies are relatively underpowered and their potential for biomarker discovery limited by the infeasibility of neural biopsy. It is therefore important to explore the potential of miRNA analysis in serum EVs (including exosomes) which originate in the brain.

Serum miRNA is protected from degradation by binding to proteins and/or encapsulation within EVs. The vesicle-associated fraction is of particular interest because the EV membrane and miRNA cargo are acquired from, and therefore representative of, the cell of origin. Neurons release EVs constitutively and in response to physiological and pathological stimuli (*9*, *10*). Proteomic analyses of neural-derived blood exosomes demonstrate that brain pathology can be detected in the periphery (*11*–*13*). EVs have been reported to transit central nervous system (CNS) and peripheral tissues (*14*, *15*) as a mechanism of intracellular communication and also functioning to promote miRNA turnover (*16*–*18*). Given that EVs express cell-of-origin markers, this suggests that serum EVs can be enriched for neuronal origin and leveraged for subsequent neuronal miRNA profiling in living subjects.

While several groups have explored the utility of L1CAM as a surface marker of neuronal EVs (*11*–*13*, *19*), recent data suggests that it may not be an optimal ligand as a proportion of circulating L1CAM exists in soluble form, in which case it may not be associated with vesicles (*20*). We observed previously that MAP1B is enriched in exosomes released from neurons (*1*). MAP1B is robustly detected in the exosome fraction from human frontal cortex and is expressed as a trans-membrane protein in neurons (*1*, *21*, *22*). We hypothesized that neuronal miRNA could be specifically profiled at a large scale in serum EVs as a quantitative trait with tissue-specific functions and serve as a window to the regulatory environment in the brain.

In this study, we immunofractionated serum EVs to obtain a neuronal-origin–enriched fraction from a large cohort of SZ cases and nonpsychiatric comparison subjects. Small RNA sequencing revealed a SZ- and disease severity–associated neuronal miRNA signature. When analyzed by cognitive subtype, SZ subjects with greater cognitive deficits (CD) were found to have a larger magnitude of miRNA dysregulation and were more likely to have severe disease [treatment-resistant schizophrenia (TRS)]. These findings provide insight into the neuronal regulatory environment in living subjects with SZ and suggest that there is profound dysregulation of miRNA associated with TRS and a CD subtype of the disorder.

## RESULTS

### Variable expression of miR-19 molecules in putative neuronal origin serum EVs

Our initial analysis sought to identify the overall miRNA profile of putative brain-associated molecules from neuronal enrichment of serum EVs, irrespective of diagnostic category (fig. S1). The miRNA profile revealed enrichment for the miR-17 ~ 92a-1 cluster (*MIR17HG*) (*P* = 3.71 × 10^−6^, Bonferroni = 2.25 × 10^−3^), the let-7 family (*P* = 9.46 × 10^−16^, Bonferroni = 5.74 × 10^−13^), miR-29 family (*P* = 1.14 × 10^−5^, Bonferroni = 6.93 × 10^−3^), and tissue specificity for the brain (cerebellum, *P* = 1.08 × 10^−16^, Bonferroni = 6.56 × 10^−14^) (table S1). Furthermore, principal components analysis (PCA) identified miR-19a and miR-19b (members of miR-17 ~ 92 cluster) as explaining the largest proportion of variation in the data (fig. S2A). Given that miR-19a and miR-19b can arise from the same primary transcript and show evidence of a positive monotonic association (but no association for the other cluster molecules; table S2), we repeated the PCA with each molecule removed and found each alone best describes the data (fig. S2, B and C).

### Differential expression of miRNA for schizophrenia and nonpsychiatric comparison subjects

**Fig. 1.**
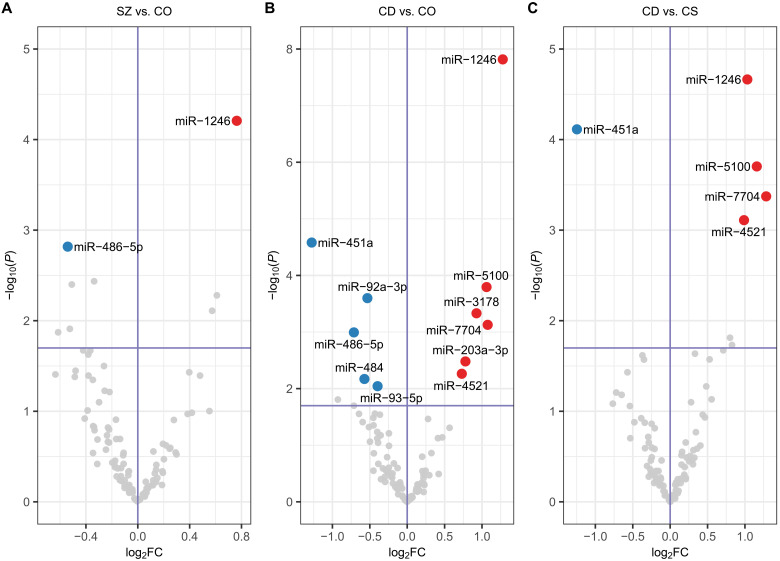
miRNA dysregulated in immunofractionated serum EVs from schizophrenia cases. (**A**) Significant differential expression (FDR < 0.1) of neuronal miRNA was observed for schizophrenia subjects (SZ) (*n* = 221) compared to nonpsychiatric comparison subjects (CO) (*n* = 256), (**B**) cognitive-deficit subtype of schizophrenia (CD) (*n* = 111) compared to nonpsychiatric comparison subjects and (**C**) CD compared to cognitively spared subtype of schizophrenia (CS) (n = 110).

We investigated the expression of miRNA in serum EVs enriched for neuronal origin. This analysis revealed two miRNAs altered [false discovery rate (FDR) < 0.1] between groups (Table 1). Specifically, miR-486-5p was observed to display reduced expression, while miR-1246 demonstrated increased expression in SZ cases compared to nonpsychiatric comparison subjects (Fig. 1A).

**Table 1.** miRNA differentially expressed in schizophrenia subjects. Cognitive deficit and cognitively spared refer to schizophrenia subjects subtyped for cognition.

Mature miRNA name	Log_2_ fold change	FDR
**Schizophrenia versus comparison**		
hsa-miR-1246	0.764	6.51E−03
hsa-miR-486-5p	−0.540	7.98E−02
**Cognitive-deficit versus comparison**		
hsa-miR-1246	1.278	1.60E−06
hsa-miR-451a	−1.277	1.38E−03
hsa-miR-5100	1.061	5.62E−03
hsa-miR-92a-3p	−0.532	6.62E−03
hsa-miR-3178	0.926	9.80E−03
hsa-miR-7704	1.076	1.31E−02
hsa-miR-486-5p	−0.713	1.52E−02
hsa-miR-203a-3p	0.778	4.34E−02
hsa-miR-4521	0.730	6.37E−02
hsa-miR-484	−0.572	7.10E−02
hsa-miR-93-5p	−0.396	8.64E−02
**Cognitively spared versus comparison**		
hsa-miR-12136	−0.620	0.350
hsa-miR-324-5p	−0.755	0.493
hsa-miR-26a-5p	−0.620	0.542
hsa-miR-146b-5p	−0.536	0.542
hsa-let-7d-3p	−0.441	0.542
hsa-miR-486-5p	−0.420	0.542
hsa-miR-425-5p	−0.441	0.542
**Cognitive-deficit versus cognitively spared**		
hsa-miR-1246	1.032	2.28E−03
hsa-miR-451a	−1.247	4.05E−03
hsa-miR-5100	1.158	6.93E−03
hsa-miR-7704	1.284	1.11E−02
hsa-miR-4521	0.988	1.63E−02
**TRS versus non-TRS (cases only)**		
hsa-miR-1246	2.038	1.08E−10
hsa-miR-5100	2.259	6.64E−08
hsa-miR-7704	2.566	4.25E−07
hsa-miR-4521	2.258	1.42E−06
hsa-miR-203a-3p	1.135	1.48E−02
hsa-miR-3178	1.252	1.48E−02
hsa-miR-199a-5p	−1.356	2.28E−02
TRS versus non-TRS plus comparison (cases and comparison)*		
hsa-miR-1246	2.185	1.80E−13
hsa-miR-5100	2.359	1.40E−10
hsa-miR-7704	2.599	1.10E−08
hsa-miR-4521	2.082	2.36E−08
hsa-miR-203a-3p	1.289	1.43E−03
hsa-miR-199a-5p	−1.263	2.33E−02
hsa-miR-3178	1.144	2.77E−02
hsa-miR-590-5p	−1.237	6.55E−02
hsa-miR-126-3p	−0.840	6.55E−02

*Adjusted for case (SZ) comparison (CO) status.

### Differential expression of miRNA in cognitive subtypes of schizophrenia cases and comparison subjects

We analyzed our data to test for differential expression of miRNA between cognitive subtypes in SZ and the nonpsychiatric comparison group, using the same pipeline and subjects as above. This analysis revealed notable differences in SZ cases with CD compared to both nonpsychiatric comparison subjects and cognitively spared (CS) schizophrenia cases. Eleven miRNAs were differentially expressed in CD compared to nonpsychiatric comparison subjects (FDR < 0.1), with six miRNAs showing increased and five miRNAs showing decreased expression (Fig. 1B). In addition, 4 of the 11 miRNAs showed an absolute fold change greater than 2.0 (Table 1). When we directly compared the CD to CS subtype of SZ, four miRNAs were observed to display increased expression while another was observed to have decreased expression (Fig. 1C). Three of the five miRNAs were observed to have an absolute fold change greater than 2.0 (Table 1). By contrast, when we compared the CS subtype with comparison subjects, we did not observe any differentially expressed miRNA, although seven molecules with reduced expression were nominally significant (*P* < 0.05) (Table 1).

### Differential expression of miRNA in TRS

**Fig. 2.**
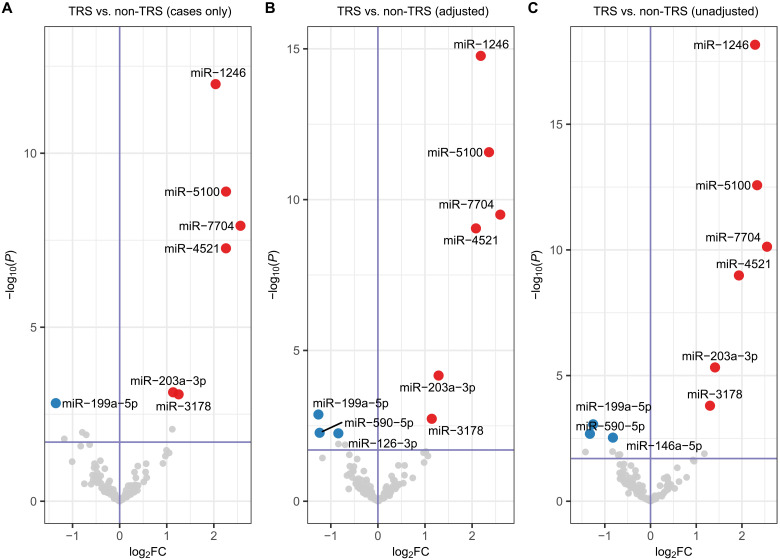
Differential expression of neuronal-origin miRNA in serum EVs from subjects with TRS. (**A**) In the cases-only analysis, significant differential expression (FDR < 0.1) of neuronal miRNA was observed for TRS (*n* = 42) compared to non-TRS (*n* = 179). (**B**) In the full cohort analysis, including nonpsychiatric comparison subjects as non-TRS, significant differential expression (FDR < 0.1) of neuronal miRNA was observed for TRS (*n* = 42) compared to non-TRS (*n* = 435, 179 cases with non-TRS and 256 nonpsychiatric comparison subjects) with adjustment (adj) and (**C**) without adjustment (unadj) for case-comparison status (SZ and CO).

We first asked if SZ cognitive subtypes (CD and CS) were disproportionately represented in severe disease, TRS and early-onset schizophrenia (EOS). While there was no overrepresentation of cognitive subtypes for EOS cases, individuals with CD were more likely than those with CS to be designated TRS (Fisher’s exact test, *P* = 0.0003, odds ratio = 4.0; 95% confidence interval, 1.8 to 9.8). In the cases-only analysis, we identified seven differentially expressed miRNA including six that were up-regulated and one down-regulated, in TRS compared to the non-TRS group (Fig. 2A and Table 1). With the exception of miR-199a-5p, the miRNAs observed to be dysregulated in the TRS group were very similar to those observed in the analyses of all SZ subjects and cognitive subtypes. To further increase the scope of severe disease–associated molecules, we repeated the analysis with nonpsychiatric comparison participants included as non-TRS. Similar to the results reported above, we identified the same six increased miRNAs in TRS (miR-1246, miR-5100, miR-7704, miR-4521, miR-203a-3p, and miR-3178) with (Fig. 2B and Table 1) and without (Fig. 2C and table S3) adjustment for case-comparison status. For miRNA with decreased expression in TRS, we again observed miR-199a-5p, in addition to miR-590-5p, whereas miR-126-3p and miR-146a-5p were decreased in the adjusted and unadjusted analyses, respectively.

There were no differences in miRNA expression between EOS and non-EOS for both cases-only and full-cohort analyses, although reduced expression of miR-3175 and miR-223-3p in cases-only EOS were nominally significant (unadjusted *P* < 0.05, table S3). Furthermore, there were no differences in miRNA expression given positive and negative symptom scores, although two and five miRNA, respectively, were nominally significant (unadjusted *P* < 0.05, table S3).

### Target gene enrichment in synaptic and neuronal biology

To gain insight into the functional significance of miRNA differentially expressed in SZ cases as a whole, as well as cognitive and TRS subtypes, we analyzed the predicted target genes and found enrichment for annotations to synaptic genes and neuronal biological processes. Specifically, in the comparison of SZ and the nonpsychiatric comparison groups, the biological process analysis displayed regulation of synapse organization and regulation of synapse structure or activity as enriched with the predicted target genes. Cellular component analysis displayed target gene enrichment in integral component of postsynaptic specialization membrane, integral component of postsynaptic membrane, postsynaptic density, asymmetric synapse, and intrinsic component of postsynaptic specialization membrane (table S4). In the comparison of CD and nonpsychiatric comparison groups, the biological process analysis displayed neuron development as enriched with the predicted target genes. Cellular component analysis revealed enrichment for these genes in synapse and postsynapse (table S5). In the comparison of CD and CS counterparts, the biological process analysis displayed generation of neurons, neurogenesis, and neuron differentiation as enriched with the predicted target genes. Cellular component analysis displayed target gene enrichment in synapse and axon (table S6). Last, in the comparison of TRS cases compared to non–treatment-resistant counterparts, the biological process analysis displayed neuron development and neuron projection development as enriched with the predicted target genes. Cellular component analysis revealed enrichment for these genes in synapse, postsynapse, and neuron projection (table S7). Gene ontology enrichment results when comparing TRS cases to all other subjects (adjusted for case/comparison status) were similar (table S8).

### Association of target gene sets with schizophrenia

**Fig. 3.**
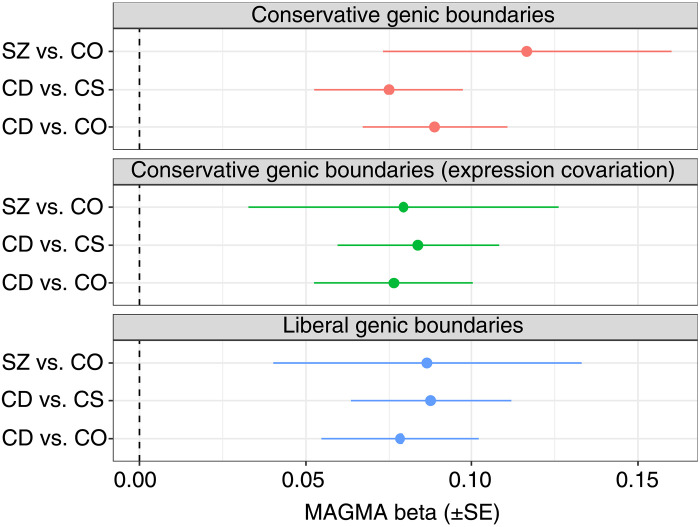
Association of gene sets from dysregulated miRNA in schizophrenia subjects. Forest plot of MAGMA gene set association results for the three sets of predicted targets from differentially expressed miRNA between groups. Each panel displays the results constructing a model using liberal or conservative definitions of the boundaries for each gene, as well as a model with conservative boundaries additionally covaried for cortical gene expression for each gene. Conservative boundaries extend the gene 5 kb upstream and 1.5 kb downstream to capture regulatory variation, while liberal boundaries are 35 and 10 kb upstream and downstream, respectively. The MAGMA β-coefficients for the gene set term are plotted with the error bars representing the standard error of the coefficient.

To further investigate the functional implications of the differentially expressed miRNA in relation to the molecular basis of disease, we tested whether their predicted targets were enriched with genes genetically associated with SZ, as indexed by genetic variants from genome-wide association study (GWAS) of the disorder. We observed that each of the three miRNA-associated gene sets displayed some evidence of association with SZ, at both conservative and liberal genic boundaries and when adjusted for cortical expression of each gene (Table 2 and Fig. 3). Of the three genesets tested using conservative genic boundaries, the SZ cases with CD compared to the group of nonpsychiatric comparison subjects were the most enriched with common-variant associations (β = 0.089, SE = 0.022, and *P* = 2.45 × 10^–5^), followed by CD compared to CS cases (β = 0.075, SE = 0.022, and *P* = 4.30 × 10^–4^) and SZ cases compared to nonpsychiatric comparison subjects, where the association was more nominal (β = 0.117, SE = 0.043, and *P* = 3.66 × 10^–3^). Among the results within the three gene sets were transcription factor 4 (TCF4, *P* = 3.45 × 10^–17^) and calcium voltage-gated channel subunit alpha1 C (CACNA1C, *P* = 4.23 × 10^–16^).

**Table 2.** Gene set association for predicted targets of differentially expressed miRNA. Conservative refers to the genic boundaries for variant to gene annotation in MAGMA, that is, gene coordinates were extended 5 kb upstream and 1.5 kb downstream to capture regulatory variation, Liberal genic boundaries were 35 kb upstream and 10 kb downstream.

Cell-type gene set	*P*_Conservative_	*P*_Liberal_
Schizophrenia vs nonpsychiatric comparison	3.66 × 10^−3^	0.03
Cognitive deficit vs cognitively spared	4.30 × 10^−4^	1.49 × 10^−4^
Cognitive deficit vs nonpsychiatric comparison	2.45 × 10^−5^	7.25 × 10^−4^

We performed a series of sensitivity analyses for the observed association signal in each miRNA target set. Covariation for cortical gene expression resulted in only a minor effect on the estimated coefficient, supporting that the relationship between these sets and SZ is not simply driven by high neurological expression of these genes (table S9). Quantile-quantile (QQ) plots of the residualized genic *Z* scores did not indicate that the association of the excitatory and inhibitory sets with SZ was driven by only a subset of genes in the set (fig. S8) (*23*). *Z* scores deviated reasonably early and consistently in the set from the plot diagonal, with the majority of observed residual *Z* values outside the upper 95% confidence band of deviation expected by chance alone. It should be noted that in all three instances, a relatively large proportion of the gene sets do not surpass the 95% confidence band, which suggests some nonuniformity in the genic association across the sets. However, given that these are large gene sets over 2000 genes each, it is unlikely that any single overlapping gene set would be responsible for the observed associations.

### Pathways enrichment of cognitive deficit miRNA

**Fig. 4.**
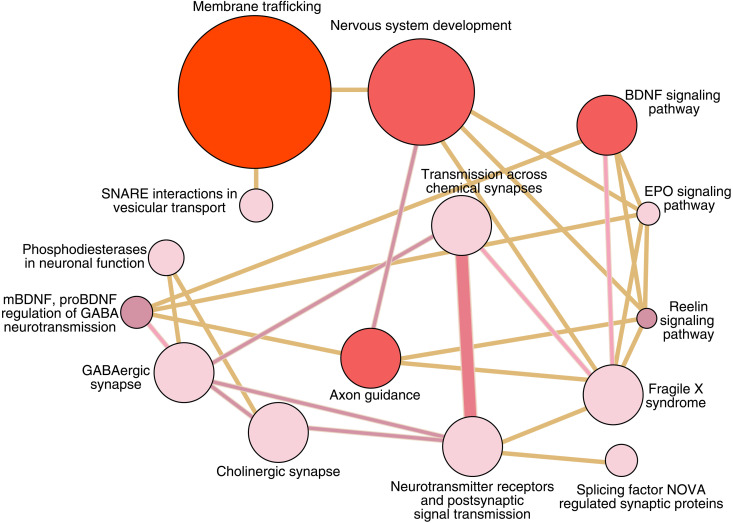
Cognitive deficit–associated miRNAs are enriched with target genes associated with neural processes. Predicted targets of miRNA dysregulated in schizophrenia cases with cognitive deficit compared to nonpsychiatric comparison subjects with binding sites for at least two miRNAs. The gene list was mapped to predefined pathways and tested (hypergeometric) for overrepresentation using Consensus Path Database. Circles (referred to as nodes) represent molecular concepts in which miRNA targets are enriched, node size represents the number of miRNA targets and node color represents the *P* value. Connected molecular concepts are represented by lines (referred to as edges) between nodes where the edge width represents a relative measure of shared molecules between concepts and edge color represents the quantity of miRNA targets that are shared between nodes. Edges are filtered to minimum relative overlap 0.08 to highlight the closest relationships between nodes and some edges omitted to improve readability, while filtered and all edges figure is available in supplementary materials (fig. S9). Among the enriched pathways are “cholinergic synapse” (*q* value = 0.024), “Nervous system development” (*q* value = 1.411 × 10^−4^), “BDNF signaling pathway” (*q* value = 1.695 × 10^−4^), “EPO signaling pathway” (*q* value = 0.005), and “Reelin signaling pathway (*q* value = 0.003).”

Given the magnitude of miRNA alterations observed in SZ cases with CD and the important role miRNA play in brain development and function, we investigated pathways predicted to be perturbed as a consequence of CD–associated miRNA. In accordance with the gene ontology analysis for the combined SZ cases, several neuronal system pathways were enriched. For example, cholinergic synapse (*q* value, 0.024), brain-derived neurotrophic factor (BDNF) signaling pathway (*q* value, 1.695 × 10^−4^), EPO (erythropoietin) signaling pathway (*q* value, 0.005), and reelin signaling pathway (*q* value, 0.003) (Fig. 4).

### Consistent differential expression of miR-1246

**Fig. 5.**
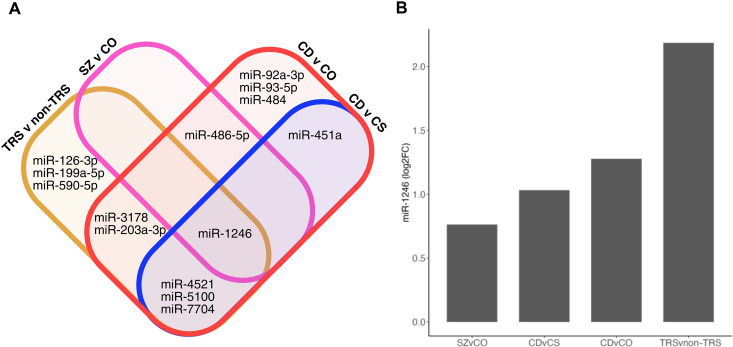
Differentially expressed miRNA within schizophrenia. (**A**) miR-1246 was the only miRNA consistently dysregulated across all contrasts (CD versus CS, CD versus CO, TRS versus nonTRS, and SZ versus CO). miR-4521, miR-5100, and miR-7704 were the next most consistently dysregulated (CD versus CS, CD versus, CO and TRS versus non-TRS), followed by miR-203a-3p and miR-3178 (CD versus CO and TRS versus non-TRS), and last, miR-451a (CD versus CS and CD versus CO) and miR-486-5p (CD versus CO and SZ versus CO). (**B**) Differential expression of miR-1246 (log_2_ fold change) across all comparisons. Schizophrenia with severe cognitive deficits (CD, *n* = 111), Schizophrenia with spared cognition (CS, *n* = 110), nonpsychiatric comparison subjects (CO, *n* = 256), TRS (*n* = 42), and subjects who are not treatment resistant include cases and comparison (non-TRS, *n* = 435).

We observed several overlapping miRNA when identifying expression differences between groups (Fig. 5A). miR-1246 was the most consistently differentially expressed, showing increased expression across all groups (Fig. 5B). High confidence predicted targets of miR-1246 are brain enriched and overlap with miR-137 targets (fig. S10, A and B). The second set of miRNAs (miR-4521, miR-5100, and miR-7704) was consistent across three groups, followed miR-203a-3p, miR-3178, miR-451a, and miR-486-5p, which were consistent across two groups.

## DISCUSSION

In this study, we explored the utility of MAP1B-immunofractionated serum EVs as a proxy of brain-associated miRNA expression and used this insight to explore the difference in serum samples from a large cohort of individuals with SZ and a nonpsychiatric comparison group. We demonstrate that enrichment for neuronal-origin serum EVs is both viable and informative for revealing a profile of miRNA species consistently observed across samples from an otherwise relatively inaccessible neural tissue. This profile was enriched for miR-17 ~ 92 cluster molecules, which are expressed in the brain and play roles in neuronal differentiation (*24*), and when overexpressed in mesenchymal stem cell exosomes, they promote neurological recovery from stroke (*25*). In addition, clustering analysis revealed that miR-19a and miR-19b (members of miR-17 ~ 92 cluster) explained the largest proportion of variation in the data. Given that these molecules play an important role in adult neurogenesis (*26*), a process affected in brain ageing (*27*), they may be indicative of ageing-associated disorders. We also showed that the brain-associated miRNA profile from all participants shows tissue specificity for the brain (cerebellum) and is enriched for let-7 and miR-29 families, molecules known to be expressed in mammalian brain (*28*, *29*). Together, this demonstrates that serum EVs enriched for neuronal origin provide a readout of the brain-associated miRNA regulatory environment in living subjects.

We investigated whether alterations in the neuronal miRNA profile could be detected in psychiatric illness and found that people with SZ do show dysregulated expression of two miRNAs, miR-486-5p and miR-1246. These two miRNAs are brain-enriched (*30*), predicted to target genes that function in synapse organization and are enriched at the postsynaptic membrane (fig. S3). In addition, miR-486-5p from L1CAM-enriched plasma EVs was recently reported to be associated with antidepressant treatment response in Major Depressive Disorder (MDD) (*19*), although L1CAM as a neuronal EV marker may not be ideal, at least in proteomics studies (*20*).

There is a broad spectrum in the symptom profile of SZ, such that biological subtypes may be more meaningful than diagnostic categories for determining mechanisms of dysfunction and improving outcomes. Cognitive symptoms are a core feature of schizophrenia and a key predictor of poor outcome, as cognitive (and negative) symptoms are not ameliorated to the same extent as positive symptoms with current pharmacotherapies and contribute to greater functional impairment (*31*, *32*). Given that miRNA play important roles in processes that subserve cognition (synaptic plasticity, learning, and memory), we investigated the neuronal miRNA profile based on cognitive subtypes (*33*). Stratification by cognition revealed subjects with more severe CD displayed an even greater degree of dysregulation in miRNA expression than was seen in CS counterparts, suggesting that the circulating neuronal miRNA profile may be informative for cognitive domains of illness. Poor outcomes are also associated with treatment resistance (*34*) and when stratified by clozapine use, the molecules associated with TRS were similar to those associated with SZ and cognitive subtypes, with the exception of miR-199a-5p, miR-126-3p, and miR-590-5p, which were decreased only in TRS. The cognitive and TRS profiles contained brain-enriched miRNA and were mostly characterized by increased expression, suggesting that the cellular compartment of origin contains an abundance of these regulatory molecules. The predicted targets of dysregulated miRNA are enriched in synaptic plasticity–related terms such as neuron development and synapse organization, while also showing enrichment for SZ-associated common variation. To the best of our knowledge, miRNA analysis of fractionated neuronal-origin EVs from the peripheral circulation of SZ patients has not been previously reported nor have MAP1B tagged EVs been collected from peripheral blood in psychiatry or other medical applications.

We observed several miRNA consistently dysregulated across groups, the most prominent being hsa-miR-1246 with increased expression in all groups. The magnitude of miR-1246 differential expression increases as we move from SZ as a whole undifferentiated group, toward more focused comparisons with severe disease (CD and TRS), suggesting that miR-1246 may be important to core features of SZ and even more so to distinct biological domains within the diagnostic category. Furthermore, we found that miR-1246 targets are specifically enriched in the brain and overlap with miR-137 targets. There is strong evidence for association between miR-137 and SZ, via risk enhancing genetic variants in the host gene (*MIR137*), enrichment of genetic variants within validated targets of miR-137 and plausible pathways in which these targets function (*35*–*38*). Given the overlap in miR-1246 and miR-137 targets, it is possible that their combined regulation is important for sculpting gene expression in pathways and networks important for neuronal function. On the other hand, hsa-miR-451a was consistently reduced in CD and this may indicate specificity to severe cognitive impairments for a subset of subjects with SZ. miR-451a was reduced in cerebrospinal fluid (CSF) exosomes from young (and late)–onset Alzheimer’s disease subjects, suggesting that miR-451a may report on cognitive decline in neurological disorders (*39*). It would be advantageous to monitor levels of these consistently mis-expressed miRNA in individuals at clinical high risk for developing SZ to identify candidate biomarkers for transition to psychotic disorders.

Our findings of SZ-associated alterations in miRNA abundance demonstrate that neuronal-origin enrichment of serum EVs reveals disease-associated miRNA profiles from disease-relevant cell types (neurons). As this may represent a niche of human brain miRNA in the peripheral circulation, we speculated that these miRNAs would have been previously observed in human CNS tissues such as CSF, prefrontal cortex, prefrontal cortex EVs, superior temporal gyrus, and amygdala. We observed that the vast majority of differentially expressed miRNA (33 of 35 unique miRNAs) have been reported as expressed in human CNS tissues and 11 of these miRNAs have been previously associated with SZ (table S10). In addition, we also queried a human brain dataset that specifically analyzed miRNA (*30*) and ranked those mature miRNAs based on the reported mean expression [counts per million (CPM) mapped miRNA reads]. We found that two of the most dysregulated miRNAs identified here (hsa-miR-486-5p and hsa-miR-92a-3p) are among the top five expressed in the human brain (fig. S4). These results support our objective to capture brain-expressed miRNA from the peripheral circulation, not unlike a recent report for MDD (*19*). Others have investigated overlap of peripherally circulating miRNA (*40*) and EV-associated miRNA (*41*) from CSF within the same subjects and report that the two biofluids have different miRNA profiles, quantitatively, and qualitatively. This suggests that enrichment for neuronal origin serum EVs may demonstrate a miRNA profile more akin to CSF (and relatively noninvasive), and as a refinement, future studies may provide validation in vivo. Moreover, just as bulk tissue analysis can dilute molecules with relatively low abundance leading to loss of biological information, our enrichment for relatively homogenous EVs rather than the global population, has revealed disease-associated miRNA that may otherwise be missed. Also, in contrast to bulk analysis of circulating EVs, the function of these miRNAs can be interpreted on the basis of the cellular context, in this case, miRNA regulation of neuronal gene expression. For example, miR-7704 is expressed at low copy number in the human brain (*30*) and this likely contributes to a lack of differential expression in bulk brain-tissue analyses, whereas in our targeted analysis, the signal-to-noise ratio is improved and we find miR-7704 to be robustly detected and up-regulated. Thus, our approach to interrogating miRNA dysregulation in an otherwise relatively inaccessible neural tissue, is highly relevant to psychiatric disorders and thus increases the scope of disease-associated molecules, an important advance given the complexity and heterogeneity of psychiatric disorders.

miRNA associated only with TRS (miR-199a-5p, miR-590-5p, and miR-126-3p) may be representative of severe disease because both human (*42*, *43*) and animal studies (*44*–*46*) collectively indicate that alterations in these miRNAs are neither drug specific or dependent. This work provides evidence that the neuronal origin miRNA profile in serum EVs may have the potential to predict TRS and could support clinical decisions to initiate clozapine treatment at an earlier stage given its advantage for treating resistant disease (*47*).

The lack of miRNA association with EOS is not unanticipated given its relatively rare occurrence (*48*), in addition to suggested differences (between EOS and adult onset SZ) in genetic and environmental contributions (*49*), both of which influence miRNA expression. Nevertheless, although decreased miR-3175 and miR-223-3p were only nominally significant in the current work, previous reports support their alterations in SZ and EOS (*50*–*52*). In particular, in the context of human neuronal and neuronal EV expression, these molecules may serve as indicators of severe disease (proxied by EOS) but larger sample sizes are needed to confirm this.

The lack of miRNA association with positive and negative symptoms is also not unanticipated given the highly heterogeneous nature of clinical presentation (*53*, *54*). There are few reports of symptom-associated miRNA alterations in SZ (*55*–*57*), so it is possible a lack of findings in other studies has led to under-reporting of this question. The previous studies reporting symptom-associated miRNA alterations all used the positive and negative symptom scale, whereas the current study used the Diagnostic Interview for Psychosis (DIP) and the Scale for the Assessment of Negative Symptoms (SANS). In addition, the current study used more than twice the number of SZ cases than the previous studies, suggesting that the current study captured a greater degree of clinical heterogeneity. It is likely that larger sample sizes, as well as consistent use of scales for the assessment of symptoms, are needed to partition patients on their symptom profiles.

Our pathways analyses indicate that targets of SZ-associated miRNA are enriched, spatially and functionally, at the synapse (fig. S3). Therefore, it is conceivable that the expulsion of miRNA, via EVs, from these discrete subcellular sites is the most efficient way to rapidly augment the local synaptic regulatory environment. In addition, when identifying pathways with respect to cognitive subtypes, EPO signaling presents an interesting finding due to brain effects, which are independent of hematopoietic effects (*58*). In the mammalian brain, EPO receptor (EPOR) mediates adult neurogenesis and neuroprotection, while cognitive challenge resulting in functional hypoxia induces up-regulation of endogenous EPO ligand and EPOR with subsequent performance improvements (*59*–*62*). Furthermore, a systematic review evaluating the efficacy of adjunctive EPO treatment found improved cognitive performance in SZ subjects (*63*). Given the importance of EPO signaling in both the brain and periphery, the data suggests that neuronal origin serum EV miRNA may represent a common language to communicate CNS conditions with distant tissues.

Last, our pathways analyses for CD miRNA targets also identified enrichment for cholinergic synapse and phosphodierterases in neuronal function, both of which are promising treatment targets for CD in SZ. A recent trial of combination cholinergics, a central agonist and peripheral antagonist at muscarinic receptors (xanomeline and trospium, respectively) for SZ patients with a clinically meaningful level of cognitive impairment, akin to our cognitive deficit group, demonstrated improvements in cognitive performance (*64*), measured by the CogState brief battery (*65*). In addition, a recent trial of the phosphodiesterase (PDE) inhibitor roflumilast (selective PDE4 inhibitor) demonstrated reduced errors in a cognitive flexibility task among SZ patients taking 100-μg dose for 8 days compared to both SZ patients taking placebo and patients taking a higher dose (250 μg) (*66*). Together, these data suggest that future clinical trials may benefit from the inclusion of miRNA from neuronal origin serum EVs, through potential to stratify patients with CD and provide a biological readout of treatment response, ultimately enabling precision medicine.

In conclusion, we developed an approach to capture an enriched fraction of neuronal-derived EVs from serum of a large cohort of individuals living with SZ. Determination of the expression of miRNA using RNA sequencing (RNA-seq) allowed the comparison of subjects with a nonpsychiatric comparison group. Given the putative role of miRNA in synaptic function, it was interesting that the difference in miRNA expression was greatest in SZ cases with severe CD when compared to either CS or nonpsychiatric comparison groups. Although medication effects, comorbidities, chronic illness, and drug use (licit and illicit) are common in SZ, these factors are difficult to accurately quantify and control, such that caution interpreting these findings is warranted. We were unable to ascertain possible differences in the time of day that blood samples were acquired and circadian rhythm modulation of miRNA expression (*67*, *68*) may have been affected, although the large number of subjects analyzed should mitigate these unknown effects. Also, it would be advantageous to replicate these findings in an independent cohort and undertake longitudinal studies with first-episode and drug-naïve SZ subjects. Nevertheless, the functional consequences of SZ-associated miRNA involve neuronal and synaptic pathways with evidence at the level of individual miRNA, the transcripts they target and genetic association with SZ. The specific mechanisms acting in this complex array of molecules, and their regulation, are unknown but the evidence suggests that the dysregulated miRNA we observed are contributing to perturbations of neurogenesis and synaptic plasticity, possibly via signaling pathways important for neuroprotection. This methodology has broad applications for the investigation of diseases that affect tissues that are difficult to biopsy, in particular those affecting the CNS.

## MATERIALS AND METHODS

### Experimental design

The present study aimed to explore the potential utility of serum EVs, enriched for neuronal origin, in the context of miRNA expression for tissues that are difficult to biopsy such as the brain. We hypothesized that the miRNA profile from individuals with SZ differs to the profile from nonpsychiatric comparison subjects. We analyzed all participants with complete data.

### Participants

Participants were drawn from the Australian Schizophrenia Research Bank (ASRB), a national research repository of phenotypic data and biological samples from English-speaking people aged 18 to 65 years (*69*). Clinical assessments at the time of ASRB enrolment included the DIP (*70*), present and lifetime substance use disorder as defined by the Diagnostic and Statistical Manual of the American Psychiatric Association (DSM-IV) and International Classification of Diseases (ICD-10) criteria (*71*), premorbid IQ (Wechsler test of adult reading) (*72*), and current IQ (Wechsler abbreviated scale of intelligence) (*73*). Neuropsychological status was assessed for immediate and delayed memory, attention, learning, and language using Repeatable Battery for Assessment of Neuropsychological Status (*74*). In addition, working memory and executive functioning were measured with letter number sequencing (*75*) and controlled oral word association test (*76*), respectively. Participants were excluded on the basis of organic brain disorder, brain injury with >24 hours postevent amnesia, IQ < 70 (full-scale), movement disorders, current diagnosis of substance dependence, and electroconvulsive therapy within last 6 months. In addition for comparison subjects, a personal or family history of psychosis or bipolar type 1 disorder led to exclusion (*69*). The current study includes 221 participants with lifetime diagnosis of a psychotic disorder and 256 nonpsychiatric comparison participants. A summary of demographic and clinical characteristics for the cohort are shown in Table 3. Refer to the Supplementary Materials (table S11) for a full description of cohort demographic and clinical characteristics. Data are available upon application to the ASRB (https://neura.edu.au/discovery-portal/asrb/). Participants in the ASRB were recruited through a national media campaign and consented to data and sample collection (2006–2011) for genomic analyses as outlined elsewhere (*69*). The use of these data was approved by the University of Newcastle Human Ethics Research Committee and all participants provided written informed consent.

**Table 3.** Summary of clinical and demographic characteristics for schizophrenia and nonpsychiatric comparison groups. Age and duration are measured in years and reported as mean ± SD. Symptom scores reported as median (interquartile range). Characteristics not applicable (N/A) to nonpsychiatric comparison subjects.

	Nonpsychiatric comparison	Schizophrenia cases	Schizophrenia cases (cognitive deficit)	Schizophrenia cases (cognitive spared)
Number of participants (%)	256 (53.7)	221 (46.3)	111 (23.3)	110 (23.1)
Male sex (%)	43.4	66.5	75.7	57.3
Age (years)	44.2 ± 13.2	40.0 ± 10.7	39.4 ± 9.9	40.5 ± 11.6
Age in years at illness onset (male/female)	N/A	23.8 ± 6.8/26.1 ± 8.5	23.8 ± 7.0/25.4 ± 7.5	23.8 ± 6.6/26.4 ± 9.0
Duration of illness in years (male/female)	N/A	15.1 ± 10.2/16.0 ± 10.3	15.0 ± 9.1/16.1 ± 9.8	15.3 ± 11.7/16.0 ± 10.6
Number of cases using at least one antipsychotic medication (%)	N/A	208 (94.1)	106 (95.5)	102 (92.7)
Number of treatment resistant cases (taking clozapine)	N/A	42	32	10
Number of early onset cases (onset before 18 years age)	N/A	31	18	13
DIP positive symptom score	N/A	13.0 (6.0)	13.0 (6.0)	13.0 (6.5)
DIP negative symptom score	N/A	2.0 (2.0)	2.0 (3.0)	1.0 (2.0)
SANS negative symptom score	N/A	25.5 (27.5)	31.0 (26.3)	16.5 (26.8)

Clustering of subjects by cognitive performance is described by Green *et al.* (*33*). In brief, grade of membership was used to determine cognitive subtype membership of SZ cases based on input variables spanning nine cognitive measures. The most parsimonious model included two classes of membership: CS subjects had low probabilities of cognitive impairment, while CD subjects showed higher probabilities of poor performance on all cognitive measures.

Positive and negative symptoms were obtained from the DIP (*70*), consistent with previous reports for this cohort (*33*, *77*). Positive symptom scores were derived using lifetime hallucination and delusion ratings, while DIP negative symptom scores were derived using restricted and blunted affect, negative formal thought disorder, social withdrawal, and lack of interest ratings for 169 SZ cases (table S11).

In addition, negative symptoms were obtained from the SANS (*78*), similar to a previous report for this cohort (*77*). SANS negative symptoms were derived from affective flattening and blunting, alogia, avolition (apathy), and anhedonia (asociality) for 168 SZ cases (table S11).

### Immunofractionation and RNA extraction

**Fig. 6.**
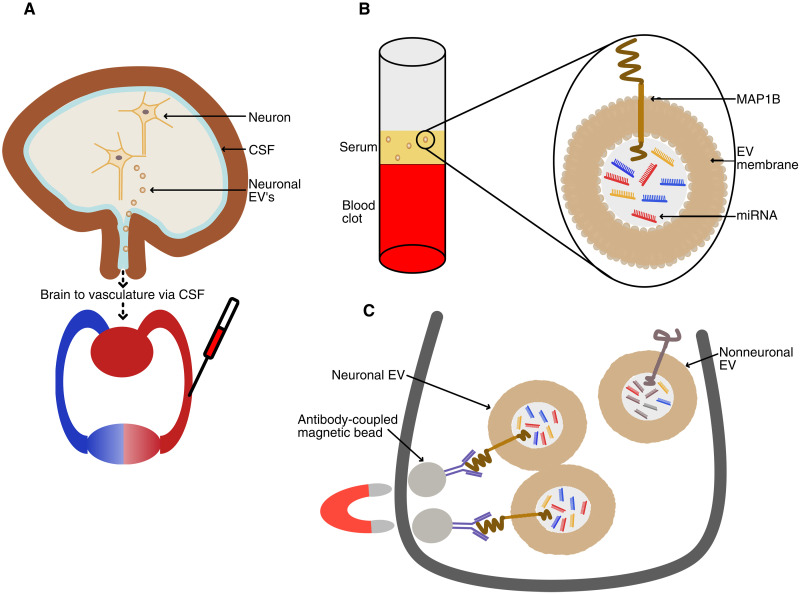
Enrichment for neuronal-origin EVs from serum. Schematic summary of immunofractionation of serum to enrich for neuronal origin EVs and subsequent extraction of neuronal RNA for small RNA sequencing. (**A**) Neurons, and other brain cell types, release EVs to the extracellular milieu both constitutively and in response to activity (refer to introduction). Released EVs, present in the interstitial fluid that surrounds neural cells, exit the brain parenchyma to reach the blood through two possible routes; the vascular pathway, guarded by the blood-brain barrier (BBB) and the cerebrospinal fluid (CSF) pathway via perivascular and perineural spaces. Given the relatively large size and negative charge of EVs, their transport to the vasculature via the BBB is uncertain, although there are reports of blood to brain transit of EVs via vesicle transcytosis (*89*). The CSF pathway is the more likely route of exit, although the specific mechanisms and relative contributions are unclear (*90*). Nevertheless, EVs have been recovered from adult human postmortem brain tissue, CSF, and peripheral biofluids (refer to discussion) and proteomics of neuron-derived plasma EVs have informed Alzheimer’s disease–related pathology (*11*–*13*). (**B**) Whole blood was obtained from study participants during clinical interview, processed to serum, and stored at −80°C. (**C**) As described in methods, serum aliquots (100 μl) were incubated with anti-MAP1B–coupled magnetic beads allowing removal of the supernatant (containing unbound EVs, including those of nonneuronal origin), while leaving an enriched fraction of relatively homogeneous neuronal origin EVs for RNA extraction and small RNA sequencing.

A schematic summary of the immunofractionation protocol is shown in Fig. 6. Although several methods for EV isolation are reported in the literature (*79*, *80*), this method was selected as the best option considering the need to balance several factors including enrichment (for neuronal origin EVs rather than all serum EVs), while minimizing sample handling and maximizing sample size and RNA recovery.

Protein G magnetic beads (50 μl) were washed three times in phosphate-buffered saline (PBS), incubated with anti-MAP1B (4 μl) (see data file S1 and fig. S5 for validation) for 1 hour at room temperature and then washed again to remove unbound antibody. One hundred microliters of serum and protease inhibitor were incubated with precoupled beads overnight at 4°C with end-on-end rotation. Following overnight incubation, serum tubes were placed on the magnetic rack to allow fractionation. The supernatant (serum depleted of EVs expressing MAP1B) was removed and immediately transferred to clean tubes and placed in −80°C storage. Meanwhile, the beads were washed three times in PBS and incubated in 100 mM glycine buffer (pH 3.0) for 3 min to release bound EVs into the supernatant. The supernatant was collected in a clean tube for immediate RNA extraction.

Total RNA was extracted from serum EVs using TRIzol, according to the manufacturer’s instructions. In brief, 1 ml of TRIzol was added to 100-μl EV supernatant. Samples were homogenized using 23-gauge needle and rested at room temperature for 5 min. Chloroform (200 μl) was added, samples vortexed, rested, and phases separated by centrifugation at 20,000*g* for 15 min at 4°C. The upper aqueous phase containing RNA was transferred to a clean tube. Isopropyl alcohol (500 μl) and RNA grade glycogen (2 μl) were added, the sample vortexed, and incubated overnight at −20°C to precipitate total RNA. The following day, samples were centrifuged at 12,000*g* for 20 min at 4°C, and the supernatant discarded. The RNA pellet was washed twice with 1 ml of ice cold 75% ethanol, air dried, resuspended in 12 μl of RNase-free water, and stored at −80°C.

### Library preparation and next-generation sequencing

Small RNA libraries were prepared using SMARTer smRNA-seq kit for illumina (Clontech Laboratories Inc.) (see Supplementary Text for detailed library preparation) according to the manufacturer’s instructions. Total RNA (7 μl) was polyadenylated, reverse transcribed, and amplified (17 cycles). Post–polymerase chain reaction clean-up was performed according to the manufacturer’s instructions. Libraries were quantified (KAPA Biosystems and Agilent smallRNA chip), equimolar-pooled, and size selected (8% acrylamide, native PAGE) by excising fragments corresponding to approximately 175 base pairs. Sequencing was performed on NovaSeq 6000 using XP workflow (two-lane flow cell) for 100 cycles of paired-end reads.

### Differential expression analysis

Raw sequencing reads were demultiplexed and quality checked using FastQC. Reads were trimmed of adapters, low-quality bases and length < 10 using Cutadapt and NGmerge. Sequencing reads were aligned using Bowtie2 and SAMtools to the human genome (GRCh38) and features counted using HTseq according to miRbase annotation of mature miRNA (v22.1). See Supplementary Text for detailed processing of sequencing data.

Differential expression of miRNA was analyzed using edgeR (*81*). Read counts were filtered to retain miRNA that reached 10 counts per million (CPM) in the smallest group size and were normalized using trimmed mean of *M* values. Under the generalized linear model framework, miRNA counts are modeled according to the negative binomial distribution and incorporate dispersion estimates with a design matrix of covariates (sequencing batch, sex, and age of participants). See Supplementary Text for detailed processing of data in edgeR.

The set of miRNA robustly detected in the whole cohort was tested for overrepresentation using TAM2.0 with background being all miRNA in the curated database. Heatmap was generated using the R package ComplexHeatmap on normalized and scaled CPM and the distance matrix ordered using the seriation package by minimizing the sum of dissimilarities. PCA was performed using FactoMineR on normalized and scaled CPM.

### miRNA target genes and functional analysis

Prediction of miRNA targets was performed with TargetScan (*82*). Human transcripts with 3′ untranslated region sequences complementary to the seed sequence of at least two miRNA were extracted. Gene lists were analyzed using the ToppFun suite with the default background gene set and the top five enriched categories were selected, sorted by Bonferroni corrected *P* value. To identify pathways enriched with CD associated miRNA targets, the gene list was submitted to Consensus Path Database and analyzed with default settings.

### Gene set association of miRNA target genes

SZ GWAS summary statistics were obtained from the schizophrenia working group of the Psychiatric Genomics Consortium for 161,405 participants, of which the majority are of European ancestry (*83*).

Gene set association of the predicted target genes of differentially expressed miRNA was tested using MAGMA (*84*), as described elsewhere (*85*). In brief, MAGMA aggregates single-nucleotide polymorphism (SNP)–wise *P* values at gene level to act as the unit of effect in a test of gene set association. Gene-based *P* values were calculated using the snp-wise = mean MAGMA model, whereby the linkage disequilibrium (LD) estimated between SNPs is leveraged to approximate the null χ^2^ distribution. We used the 1000 genomes phase 3 European reference panel to provide the LD estimations. Autosomal gene coordinates in hg19 assembly were obtained from National Center for Biotechnology Information and the 1000 genomes phase 3 European panel used as an LD reference. Genes within the MHC region were not considered due to the haplotype complexity of that region, as is usual practice (*86*). We extended the boundaries of the genic coordinates upstream and downstream to capture regulatory variation with both a conservative [5 kilobases (kb) upstream and 1.5 kb downstream] and liberal genic (35 kb upstream and 10 kb downstream) boundary definition used for analyses.

After probit transformation of gene-based *P* to *Z*, MAGMA constructs a linear regression model such that *Z* was the outcome and a binary indicator of gene set membership an explanatory variable to test whether genes in the set were more associated than all other genes considered. This model was covaried for confounders, including gene size and minor allele count (*87*). Given that the gene sets considered are highly brain expressed, we additionally constructed a model covaried for the cortical expression of each gene (median transcript per million, PsychENCODE dorsolateral prefrontal cortex dataset) to assess whether this may bias the estimated association (*23*, *86*, *88*). Furthermore, we generated QQ plots to visualize expected and observed residual genic *Z* scores from a null model without the gene set of interest, as outlined elsewhere (*23*). The plot should start to deviate from the diagonal early and consistently if the association is not driven by a subset of genes in the set. An upper, one-sided 95% confidence band was plotted which represents deviation from the plot diagonal by chance.

### Statistical analyses

Statistical analyses were performed in *R* software (version 4.0) using RStudio (version 2022.02.3 + 492). Differential expression of miRNA was determined with edgeR package (version 3.34.0) using the likelihood ratio test and adjustment for multiple testing of raw *P* values by FDR. Differential expression of miRNA was deemed significant when adjusted *P* < 0.10. Fisher’s exact test for count data (two-sided) was used to determine the presence of a relationship between cognitive subtypes and treatment resistance in schizophrenia. Gene set association as implemented in MAGMA is described in methods. *P* values from functional enrichment analyses were adjusted for multiple comparisons by Bonferroni correction (ToppFun) and FDR method (Consensus Path Database, reported as *q* value) and deemed significant when adjusted *P* < 0.05.

## Supplementary Materials

### This PDF file includes:

Supplementary text Figs. S1 to S10 Tables S1 to S9 Legends for tables S10 and S11 References
