# Supplementary material for: miRNA cargo in circulating vesicles from neurons is altered in individuals with schizophrenia and associated with severe disease
Source: Sci Adv. 2023 Nov 29;9(48):eadi4386. doi: 10.1126/sciadv.adi4386 (PMC10686555; doi:10.1126/sciadv.adi4386)
Supplement: Supplementary file 1 — Supplementary text Figs. S1 to S10 Tables S1 to S9 Legends for tables S10 and S11 References [file sciadv.adi4386_sm.pdf]

Science Advances
AAAS

Supplementary Materials for

miRNA cargo in circulating vesicles from neurons is altered in individuals with schizophrenia and associated with severe disease

Michelle M. Barnett *et al.*

Corresponding author: Murray J. Cairns, murray.cairns@newcastle.edu.au

Sci. Adv. **9**, eadi4386 (2023)
DOI: 10.1126/sciadv.adi4386

The PDF file includes:

Supplementary text
Figs. S1 to S10
Tables S1 to S9
Legends for tables S10 and S11
References

Other Supplementary Material for this manuscript includes the following:

Tables S10 and S11

Supplementary text

Antibody validation

Rabbit anti-MAP1B antibody (catalogue number A301-446A) was obtained from Bethyl Laboratories, trading as Fortis Life Sciences. Product information can be viewed at https://www.fortislife.com/products/primary-antibodies/rabbit-anti-map1b-antibody/BETHYL-A301-446 while validation information can be viewed at https://www.fortislife.com/antibody-validation. Invitrogen Dynabeads Protein G magnetic beads were obtained from Life Technologies Australia (catalogue number 10004D). Product information can be viewed at https://www.thermofisher.com/order/catalog/product/10004D.
In addition to the manufacturer's antibody validation, we further demonstrated the competency of our protocol in the context of anti-MAP1B coupled magnetic beads to recover RNA from putative neuronal-origin extracellular vesicles (EVs) in human serum. Firstly, we tested whether pre-clearing serum affected RNA recovery. Specifically, serum was incubated with naked beads (nil anti-MAP1B) to allow non-specific binding of serum proteins to be retained on-bead and supernatant (pre-cleared serum) recovered. Then, using fresh antibody-coupled magnetic beads incubated with pre-cleared serum or serum that was not pre-cleared demonstrated equivalent RNA recovery (7.9ng/µL and 8.0ng/µL, respectively) (determined by NanoDrop), suggesting non-specific binding to protein G magnetic beads had no effect on RNA recovery from neuronal-origin serum EVs. Secondly, we observed >15-fold recovery of RNA when comparing our protocol with and without anti-MAP1B (fig. S5), suggesting the antibody is fit for purpose.

Small RNA sequencing

Small RNA libraries were prepared using the SMARTer smRNA-Seq Kit for Illumina, according to the manufacturer's instructions. For each sample, 7µL of total RNA was polyadenylated via the addition of 3µL Polyadenylation Master Mix and incubation at 16°C for 5 min, after which samples were cooled at 4°C for 1 min. For reverse transcription, 1µL of 3′ smRNA dT primers was added and samples were incubated at 72°C for 3 min then cooled at 4°C for 2 min, followed by addition of 9µL Reverse Transcription Master Mix and incubated with the following program: 42°C 1 hour, 70°C 10 min, 4°C hold. Library amplification and addition of SMARTer unique dual indexes (UDI) was performed by adding 76µL of PCR Master Mix, 4µL of each UDI and incubating samples under the following conditions:

- 98°C for 1 min.
- 17 cycles of:
  - 98°C for 10 sec.
  - 60°C for 5 sec.
  - 68°C for 10 sec.
- 4°C final hold.

Library purification was performed with a NucleoSpin Gel and PCR Clean-Up kit as per the manufacturer's instructions. Briefly, PCR reactions were combined with 2 volumes (i.e. 200µL) Buffer NTI, transferred to a DNA-binding NucleoSpin Gel and PCR Clean-up column and bound to the silica membrane via centrifugation (1 min, 3000 x g). The membrane was then washed twice with 4mL Buffer NT3 and dried via centrifugation (10 min, 3000 x g). To elute cDNA, 30µL Buffer NE was added, after which samples were incubated at 70°C for 5 min and

centrifuged (2 min, 3000 x g). All libraries were subsequently quantified using Agilent High Sensitivity DNA chips and a KAPA Library Quantification Kit for Illumina Platforms, pooled in equimolar ratios and volumes and size selected via 8% native acrylamide PAGE. The gel was incubated with SYBR gold nucleic acid gel stain and bands of ~175bp, corresponding to microRNA, were excised. Size selected cDNA was eluted and quantified using Agilent High Sensitivity DNA chips and a KAPA Library Quantification Kit. Sequencing libraries, prepared according to the NovaSeq XP workflow, were diluted to a final loading concentration of 200pM using 10mM Tris-HCl pH 8.5 and denatured with 0.2N NaOH. Paired-end sequencing for 101 cycles was performed using the NovaSeq6000 platform.

Raw small RNA bcl sequencing files were demultiplexed and converted to fastq format using *bcl2fastq* (v.2.20; Illumina) with automatic adapter trimming disabled. Data quality was assessed using *FastQC* (v0.11.8). *Cutadapt* (v2.6) was used to remove sequencing adapters and trim the reads of low quality bases. Specifically, 3 nucleotides and 14 nucleotides were removed from the 5' end of read 1 and 2, respectively for all reads. Poly-A tails from the 3' end of read 1 and poly-T tails from the 5' end of read 2 were removed, and any remaining low-quality reads were removed from the 3' ends of reads, using a threshold Phred33 score of 28. Additionally, using *NGmerge* (v0.3) in adapter-removal mode, 3' overhangs and unmatched bases were removed. To ensure good quality reads >10bp long, a final trimming (*Cutadapt*) step, as described above, was repeated. Reads were next aligned to the human (*Homo sapiens*) reference genome (GRCh38 assembly, NCBI) with *bowtie2* (v2.4.1) default settings and sorted and indexed with SAMtools. Next, reads were counted with *HTSeq* (v0.9.1), using the human (*Homo sapiens*) reference (hsa.gff3) from miRbase v22.1 to define mature miRNA features. *HTSeq* was run in "union" mode, its strandedness option was set to forwards, and a minimum mapping quality score threshold of 1 was used. Unaligned and ambiguous reads were removed, and count files were merged into a single matrix, with rows representing miRNAs and columns representing samples. The count matrix was imported into *R* and analysed for differential expression with *EdgeR* (v3.34.0) (*90*). Four differential expression comparisons were made: all schizophrenia cases vs. all comparison subjects; cognitive deficit subtype of schizophrenia vs. all comparison subjects; cognitive spared subtype of schizophrenia vs. all comparison subjects and cognitive deficit subtype of schizophrenia vs. cognitive spared subtype of schizophrenia. The count matrix was filtered to only retain libraries that summed to at least 1000 counts, followed by filtering to retain miRNAs that reached 10 counts per million (CPM) in the smallest group (221 schizophrenia cases). Note, to keep analyses consistent, we used this filtering step for both the full cohort analysis and the subtype analyses. Data was then normalised and dispersions were estimated using *edgeR*'s *calcNormFactors* and *estimateDisp* functions, respectively. At each stage, MDS and BCV plots were used to visually inspect the data for quality control issues. Finally, differential expression was assessed using *edgeR*'s likelihood ratio test method.

Code for processing of small RNA sequencing data and differential expression as follows;

1. <u>Remove adapters (-a, -A) and poor quality bases (-u, -U)</u>
   cutadapt -a AGATCGGAAGAGCACACGTCTGAACTCCAGTCA -A AGATCGGAAGAGCGTCGTGTAGGGAAAGAGTGT -u 3 -U 14 -o ./<sample>_R1_001.cut1.fastq.gz -p ./<sample>_R2_001.cut1.fastq.gz ./<sample>_R1_001.fastq.gz ./<sample>_R2_001.fastq.gz

2. Remove polyA and polyT (-a, -G), high quality polyG trim (due to NovaSeq 2-colour chemistry, --nextseq-trim), length trim (-m)

```
cutadapt -a "A{100}" -G "T{100}" --nextseq-trim=28 -m 10 -o
./<sample>_R1.cut2.fastq.gz -p ./<sample>_R2.cut2.fastq.gz
./<sample>_R1.cut1.fastq.gz ./<sample>_R2.cut1.fastq.gz
```

3. Remove 3' overhangs

```
NGmerge -m 10 -a -e 10 -z -o ./<sample>_R1_001.merge.1.fastq.gz
./<sample>_R2_001.merge.1.fastq.gz -1 ./<sample>_R1_001.cut2.fastq.gz -2
./<sample>_R2_001.cut2.fastq.gz
```

4. Repeat step 2 to ensure good quality

```
cutadapt -a "A{100}" -G "T{100}" --nextseq-trim=28 -m 10 -o
./<sample>_R1.cut3.fastq.gz -p ./<sample>_R2.cut3.fastq.gz
./<sample>_R1_001.merge.1.fastq.gz ./<sample>_R2_001.merge.1.fastq.gz
```

5. Map reads (-x), index (-b) and sort

```
bowtie2 -x ./Homo_sapiens/NCBI/GRCh38/Sequence/Bowtie2Index/genome -1
./<sample>_R1.cut3.fastq.gz -2 ./<sample>_R2.cut3.fastq.gz -S
./<sample>_aln_grch38.sam samtools view -b -h ./<sample>_aln_grch38.sam >
<sample>_aln_grch38.bam
samtools sort -o ./<sample>_sorted_aln_grch38.am > <sample>_aln_grch38.bam
```

6. Count reads

```
htseq-count -t miRNA -i Name -m union -s yes -f bam -a 1 ./<sample>_sorted
_aln_grch38.bam /hsa.gff3 > ./count.<sample>.txt
```

7. Differential expression using *edgeR* package

```
# Import edgeR
library(edgeR)

# Read in counts
b1counts <- read.delim("batch1.merged.counts.txt", row.names = 1)
b2counts <- read.delim("batch2.merged.counts.txt", row.names = 1)
b3counts <- read.delim("batch3.merged.counts.txt", row.names = 1)
b4counts <- read.delim("batch4.merged.counts.txt", row.names = 1)

# Remove low count libraries
b1counts <- b1counts[colSums(b1counts)>1000]
b2counts <- b2counts[colSums(b2counts)>1000]
b3counts <- b3counts[colSums(b3counts)>1000]

b4counts <- b4counts[colSums(b4counts)>1000]

# Combine counts for all batches
allCounts <- cbind(b1counts, b2counts, b3counts, b4counts)
```

```
# Read in phenotype data and define groups (SZ = schizophrenia, CO = comparison
subjects, szCD = cognitive deficit subtype schizophrenia, szCS = cognitive spared
subtype schizophrenia)
pheno <- read.csv("phenotype.csv", stringsAsFactors = FALSE)
sampleNames <- colnames(allCounts)
groups_SZ_CO <- pheno[sampleNames,]$bi_phenotype
groups_szCD_szCS_CO <- pheno[sampleNames,]$multi_phenotype

# Read in ASRB demographics
asrb_full <- read.csv("ASRB_spss_copy_n477.csv", stringsAsFactors = FALSE)
asrb_full <- asrb_full[!duplicated(asrb_full$id),]
rownames(asrb_full) <- paste("id", asrb_full$id, sep = "")

# Join pheno and ASRB
asrb_pheno <- left_join(mypheno, asrb_full, by = "sample")

# Assemble positive and negative symptom variables
# sans_neg=select variables of interest (twenty-two SANS items; sans-affect-
facial_expression, sans-affect-spontaneous_movement, sans-affect-gestures, sans-affect-
eye_contact, sans-affect-non_responsivity, sans-affect-inappropriate, sans-affect-
vocal_inflections, sans-affect-global_rating, sans-alogia-poverty_speech, sans-alogia-
content_speech, sans-alogia-blocking, sans-alogia-latency_response, sans-alogia-
global_rating, sans-avolition-grooming, sans-avolition-impersistence, sans-avolition-
physical_aergia, sans-avolition-global_rating, sans-asociality-recreational, sans-
asociality-sexual, sans-asociality-intimacy, sans-asociality-relationships, sans-asociality-
global_rating.
# dip_neg=dip90,91,97 and 436(socialwithdrawal) and 466(socialfunctionInterests)
# select variables of interest for positive symptoms, lifetime only
# dip_pos=hallucinations (dip49 - dip53), delusions (dip58 - dip64)
# six items for dip49lt and one item each for dip50-53, dip58-64 (seventeen items total).

sans_neg <- as.data.frame(asrb_pheno[,c(1:6,891:912)])
rownames(sans_neg) <- sans_neg$sample

dip_neg <- as.data.frame(asrb_pheno[,c(1:6,871,872,878,436,466)])
rownames(dip_neg) <- dip_neg$sample

dip_pos <-
as.data.frame(asrb_pheno[,c(1:6,648,651,654,657,660,663,666,669,672,675,690,693,696,
699,702,705,708)])
rownames(dip_pos) <- dip_pos$sample

# keep cases
sans_neg_cases <- sans_neg %>% filter(bi_pheno == "SZ")
dip_neg_cases <- dip_neg %>% filter(bi_pheno == "SZ")
dip_pos_cases <- dip_pos %>% filter(bi_pheno == "SZ")
```

```
# make symptom sum for dip and sans
sans_neg_cases$sum_sans_neg <- rowSums(sans_neg_cases[,c(7:28)])
dip_neg_cases$sum_dip_neg <- rowSums(dip_neg_cases[,c(7:11)])
dip_pos_cases$sum_dip_pos <- rowSums(dip_pos_cases[,c(7:23)])

# make 2 symptoms objects, dip=dip_pos and dip_neg, sans=dip_pos and sans_neg
myjoin_dip <- full_join(dip_pos_cases, dip_neg_cases, by = "sample")
myjoin_sans <- full_join(sans_neg_cases, dip_pos_cases, by = "sample")

# omit cases with NA in any variable
myjoin_dip_complete <- na.omit(myjoin_dip)
myjoin_sans_complete <- na.omit(myjoin_sans)

# Found sum_dip_neg error for sample 250 in Table S11; coded 88 as uncertain for lack
of interest, remove sample 250 as they are essentially NA.
myjoin_dip_complete <- myjoin_dip_complete[-119,]

# Create DGEList objects
y <- DGEList(counts = allCounts, group = groups_SZ_CO)
y.sub <- DGEList(counts = allCounts, group = groups_szCD_szCS_CO)

# Create MDS plots (SZ, CO)
cn.col="blue"
sz.col="brown"
cols=groups_SZ_CO
cols[cols=="SZ"]=sz.col
cols[cols=="CO"]=cn.col
plotMDS(y,labels=colnames(y$counts),col=cols,las=1)

# Create MDS plots (szCD, szCS, CO)
cn.col="blue"
cs.col="brown"
cd.col="khaki"
cols=groups_szCD_szCS_CO
cols[cols=="szCD"]=cd.col
cols[cols=="szCS"]=cs.col
cols[cols=="CO"]=cn.col
plotMDS(y.sub,labels=colnames(y.sub$counts),col=cols,las=1)

# Define threshold for miRNA counts and smallest groups
threshold_SZ_CO = ceiling(10/(max(y$samples$lib.size)/1000000))
numSz = sum(groups_SZ_CO=="SZ")
numCo = sum(groups_SZ_CO=="CO")
minGroup = min(c(numSz, numCo))
threshold_szCD_szCS_CO = ceiling(10/(max(y.sub$samples$lib.size)/1000000))
```

````
numszCD = sum(groups_szCD_szCS_CO=="szCD")
numszCS = sum(groups_szCD_szCS_CO=="szCS")
numCo = sum(groups_szCD_szCS_CO=="CO")
minGroup.sub = min(c(numszCD, numszCS, numCo))

# Apply filter to retain miRNAs above threshold in smallest group
keep <- rowSums(cpm(y)>threshold_SZ_CO) >= minGroup
y2 <- y[keep,,keep.lib.sizes=FALSE]
keep <- rowSums(cpm(y.sub)>threshold_szCD_szCS_CO) >= minGroup.sub
y2.sub <- y.sub[keep,,keep.lib.sizes=FALSE]

# Normalise library sizes
y3 <- calcNormFactors(y2)
y3.sub <- calcNormFactors(y2.sub)

# subset y3 for complete cases in pos and neg symptoms
rownames(mycases_dip_complete) <- mycases_dip_complete$sample
y3_169 <- y3[, intersect(rownames(mycases_dip_complete), rownames(y3$samples))]
rownames(mycases_sans_dip_complete) <- mycases_sans_dip_complete$sample
y3_168 <- y3[, intersect(rownames(mycases_sans_dip_complete),
rownames(y3$samples))]

# create design matrix, with sum pos and neg symptoms
#mydesign <- mycases_dip_complete[,c(2:4,6:8)]
mydesign <- mycases_sans_dip_complete[,c(2:4,6:8)]
```
# convert batch, sex and pheno to factors and relevel
mynames <- c('batch.x', 'sex.x', 'GoM_pheno.x')
mydesign[,mynames] <- lapply(mydesign[,mynames], factor)
mydesign$GoM_pheno.x <- relevel(mydesign$GoM_pheno.x, ref = "CS")

# Read in covariates, create design matrix and estimate dispersions
d <- read.delim("covariates.txt")
design <- model.matrix(~ batch + sex + age + phenotype_SZ_CO, data = d)
y4 <- estimateDisp(y3, design)

design.sub <- model.matrix(~ batch + sex + age + phenotype_szCD_szCS_CO, data = d)
y4.sub <- estimateDisp(y3.sub, design.sub)

design_169 <- model.matrix(~ batch.x + sex.x + age.x + sum_dip_neg + sum_dip_pos,
data = mydesign)
y4_169 <- estimateDisp(y3_169, design_169)

design_168 <- model.matrix(~ batch.x + sex.x + age.x + sum_sans_neg + sum_dip_pos,
data = mydesign)
y4_168 <- estimateDisp(y3_168, design_168)
````
````

```
# Fit generalised linear models
fit <- glmFit(y4, design)

fit.sub <- glmFit(y4.sub, design.sub)

fit_169 <- glmFit(y4_169, design_169)

fit_168 <- glmFit(y4_168, design_168)

# Test for miRNA differential expression using likelihood ratio test (LRT)
lrt_BvA <- glmLRT(fit, coef = 2)
lrt_CvA <- glmLRT(fit, coef = 3)
lrt_DvA <- glmLRT(fit, coef = 4)
lrt_MvF <- glmLRT(fit, coef = 5)
lrt_age <- glmLRT(fit, coef = 6)
lrt_SZvCO <- glmLRT(fit, coef = 7)
lrt_BvA <- glmLRT(fit.sub, coef = 2)
lrt_CvA <- glmLRT(fit.sub, coef = 3)
lrt_DvA <- glmLRT(fit.sub, coef = 4)
lrt_MvF <- glmLRT(fit.sub, coef = 5)
lrt_age <- glmLRT(fit.sub, coef = 6)
lrt_szCDvCO <- glmLRT(fit.sub, coef = 7)
lrt_szCSvCO <- glmLRT(fit.sub, coef = 8)
lrt_szCDvszCS <- glmLRT(fit.sub, coef = 9)

lrt_BvA <- glmLRT(fit.sub, coef = 2)
lrt_CvA <- glmLRT(fit.sub, coef = 3)
lrt_DvA <- glmLRT(fit.sub, coef = 4)
lrt_MvF <- glmLRT(fit.sub, coef = 5)
lrt_age <- glmLRT(fit.sub, coef = 6)
lrt_szCDvszCS <- glmLRT(fit_169, coef = 7)
lrt_dip_pos <- glmLRT(fit_169, coef = 8)
lrt_dip_neg <- glmLRT(fit_169, coef = 9)

lrt_dip_pos <- glmLRT(fit_168, coef = 8)
lrt_sans_neg <- glmLRT(fit_168, coef = 9)

# coefficients 2,3,4 refer to sequencing batches, coefficient 5 refers to sex, coefficient 6
refers to age, coefficients 7,8,9 refer to phenotype or symptom scores.
```

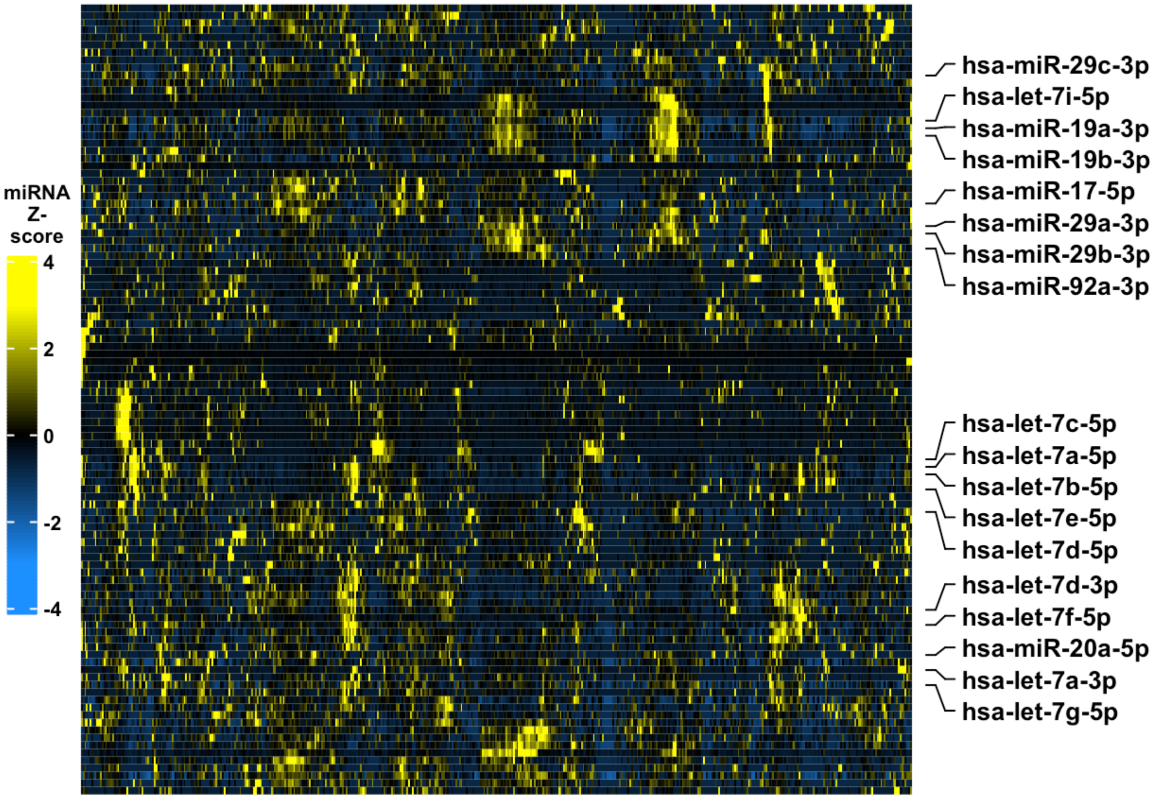

Figure S1. Heat map of neuronal origin miRNA consistently observed across samples. The expression of each miRNA (n=105) in each sample (n=477) is represented by a square. Yellow = high expression, blue = low expression. Sample labels in the heat map are omitted due to the large number of samples. Labels for miRNA in the heat map are limited to those miRNA identified from the enrichment analysis (table S2), irrespective of psychiatric phenotype, and include miR-17 cluster, let-7 family and miR-29 family. miRNA expression is normalised counts per million, converted to z-score. Heat map constructed by ordering the distance matrix to minimize the Hamilton path length.

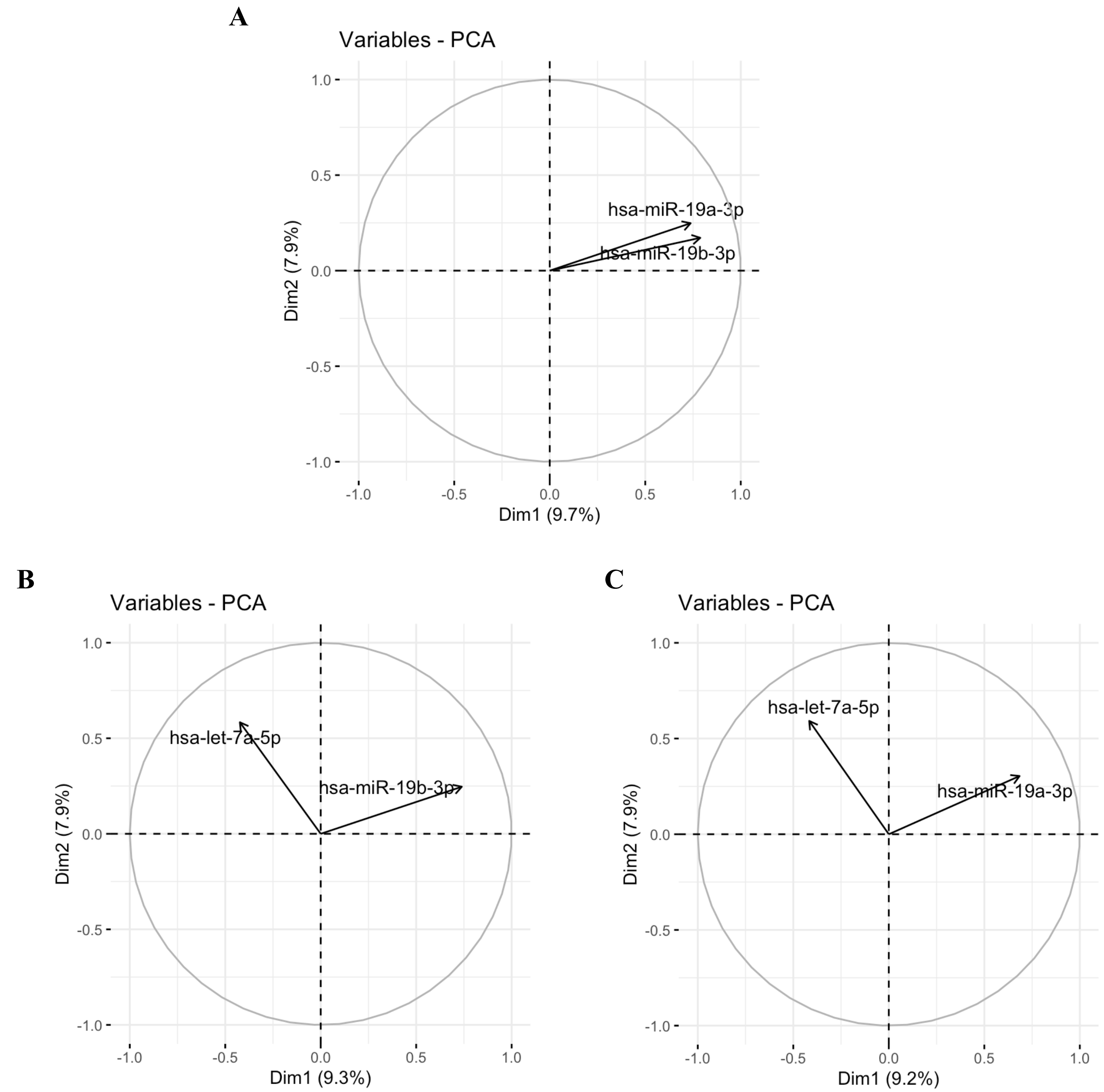

Figure S2. Principal component analysis of neuronal origin miRNA from serum extracellular vesicles. Expression of miRNA (normalised counts per million, scaled) in the full cohort (n=477) reveals (**A**) hsa-miR-19b-3p and hsa-miR-19a-3p explain the most variation in dimension one, represented as the highest quality variables on the factor map by cos2 (squared coordinates). Given that hsa-miR-19b-3p and hsa-miR-19a-3p are transcribed from the same primary transcript and show evidence of correlated expression (table S8), we repeated PCA (**B**) without hsa-miR-19a-3p and then (**C**) without hsa-miR-19b-3p, in each case showing a small reduction in proportion of variance retained in dimension one.

A B

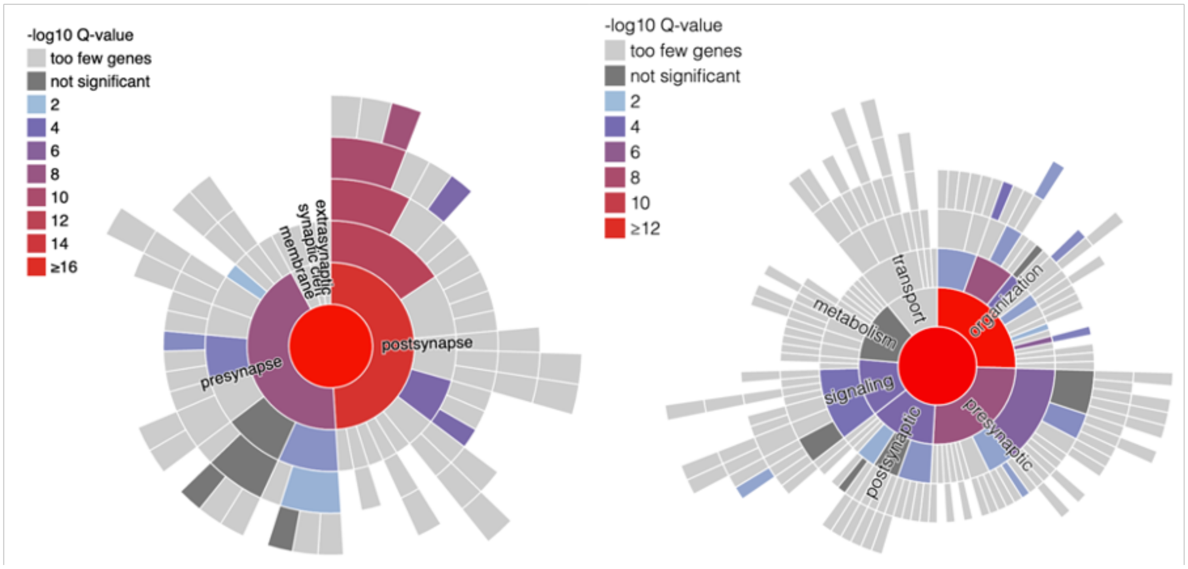

Figure S3. Schizophrenia associated miRNA targets are enriched at the synapse. (**A**) Cellular component analysis identified the predicted target genes of miRNA differentially expressed in schizophrenia subjects are overrepresented at postsynaptic sites and (**B**) function in synapse organization.

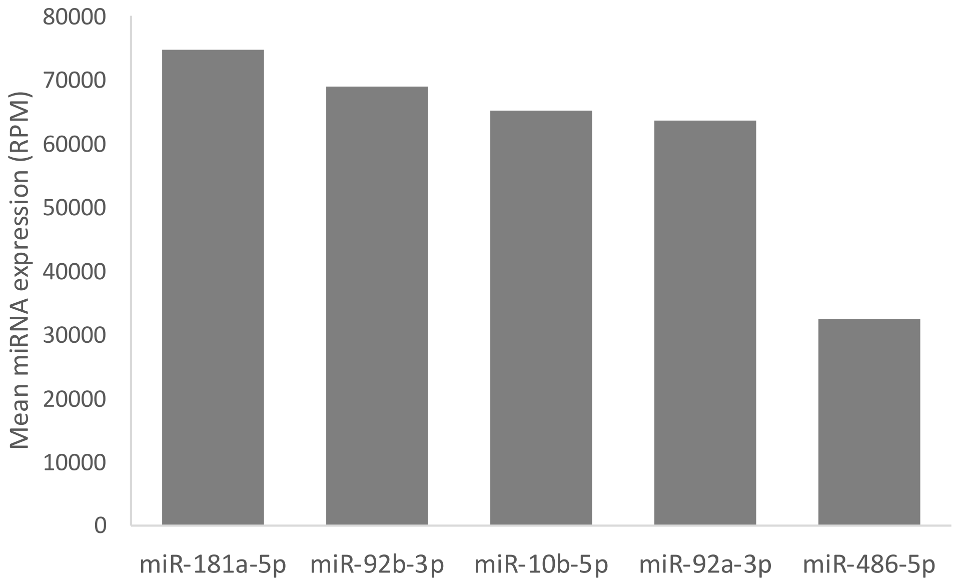

Figure S4. Schizophrenia associated miRNA from neuronal origin serum extracellular vesicles are highly expressed in the human brain. The five most highly expressed miRNA in human brain (miRmine, average Reads per Million (RPM)) include miR-92a-3p and miR-486-5p, both of which are significantly reduced in cognitive deficit subtype schizophrenia while miR-486-5p is significantly reduced in the combined schizophrenia group. Note, for space reasons, “hsa-“ has been dropped from miRNA names.

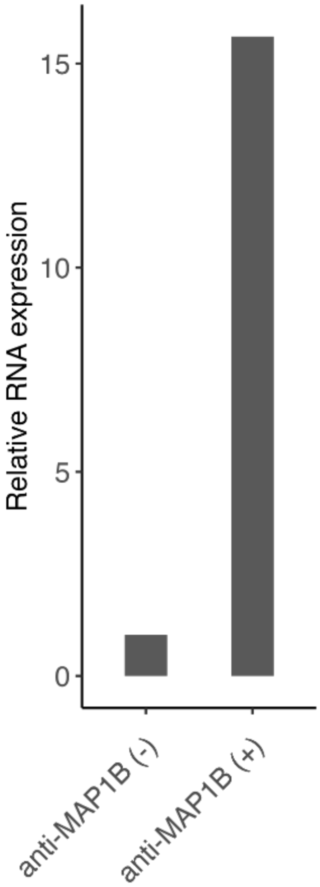

Figure S5. Validation of anti-MAP1B. Relative RNA expression increased > 15-fold when anti-MAP1B coupled beads (“anti-MAP1B (+)”) were used to enrich for neuronal origin serum EVs compared to nil antibody control (“anti-MAP1B (-)”).

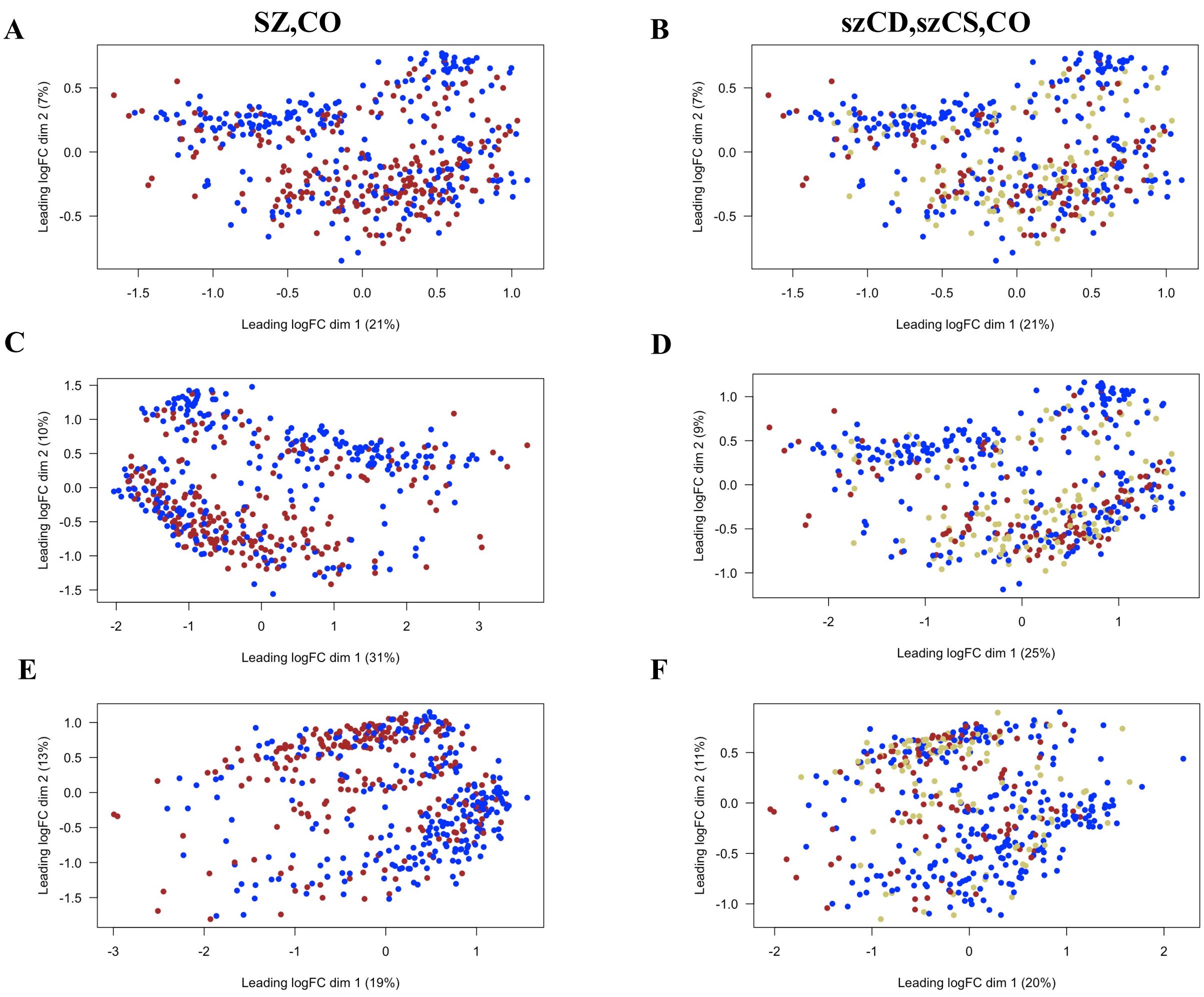

Figure S6. Processing small RNA sequencing data. (**A,B**) Multidimensional scaling (MDS) plots for miRNA before filtering, (**C,D**) after filtering to remove low count reads and (**E,F**) following normalization. (**A,C,E**) Left panels are miRNA leading log fold changes between each pair of samples with respect to schizophrenia (brown) versus comparison subjects (blue) while (**B,D,F**) right panels are miRNA leading log fold changes between each pair of samples with respect to cognitive deficit subtype schizophrenia (khaki) cognitive spared subtype schizophrenia (brown) and comparison subjects (blue). SZ=schizophrenia, CO=comparison, szCD= cognitive deficit subtype schizophrenia, szCS= cognitive spared subtype schizophrenia.

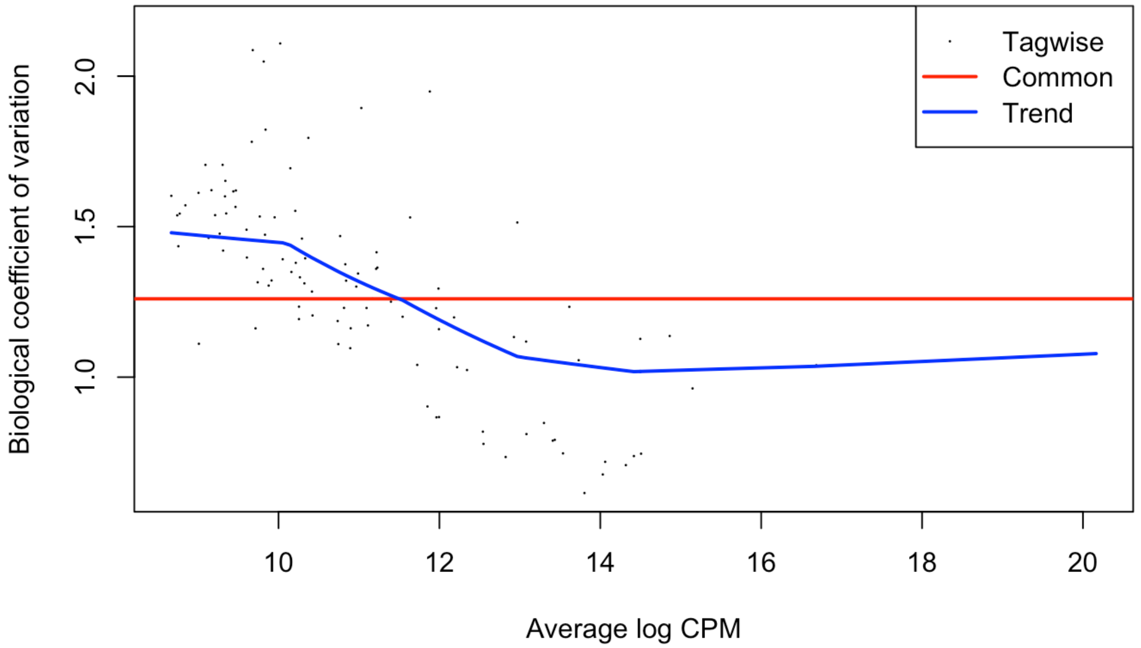

Figure S7. Estimating biological variation. Plot showing biological coefficient of variation (BCV) against average log_2 expression (CPM) for each miRNA, after normalization. As expected, dispersion estimates are high for low abundance miRNA and decrease as expression increases.

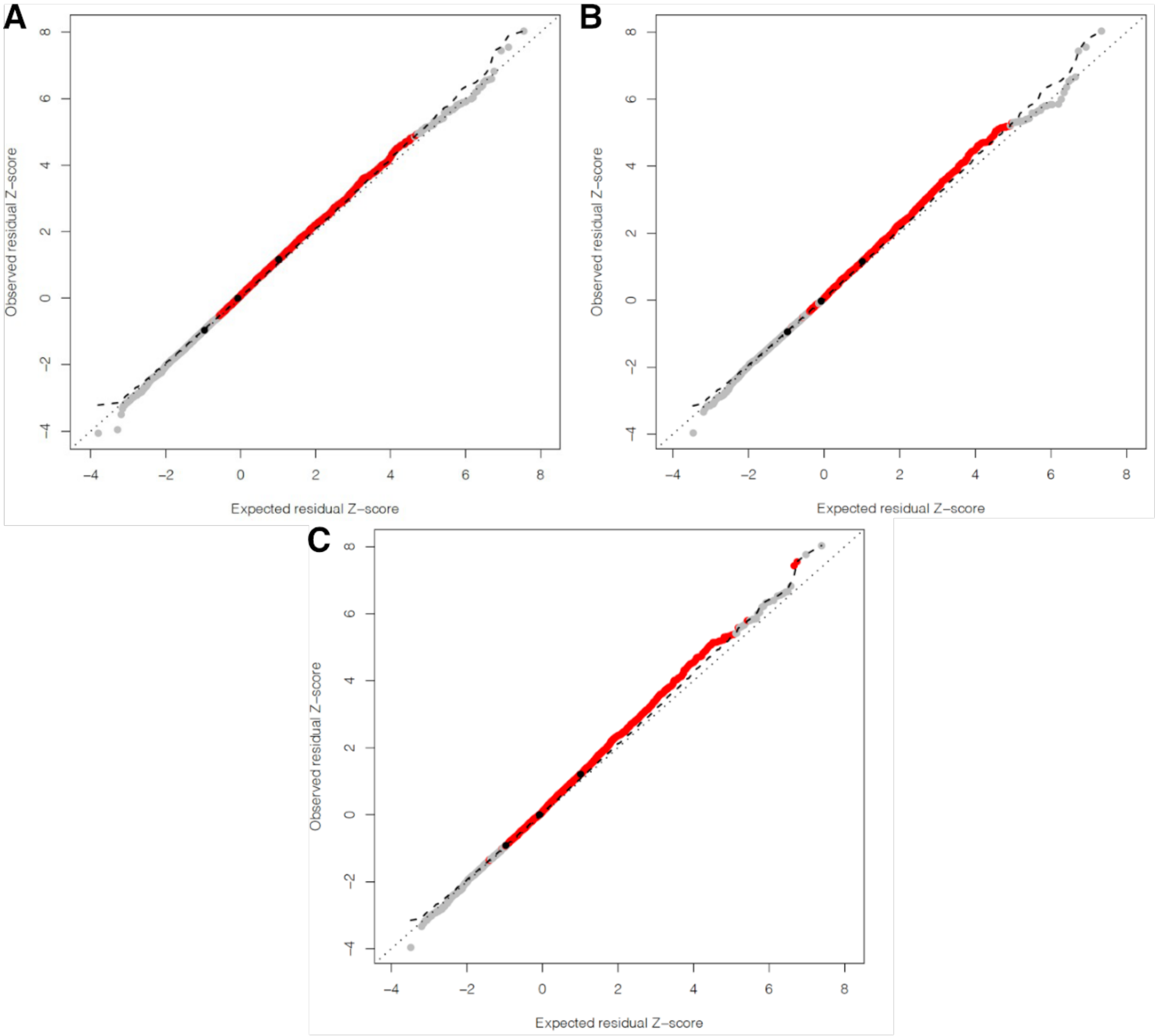

Figure S8 miRNA target gene-set association with schizophrenia risk. QQ plots of residualized genic Z values do not indicate that association of excitatory and inhibitory sets with schizophrenia (SZ) was driven by only a subset of genes in the set. (**A**) Targets of miRNA differentially expressed in SZ compared to non-psychiatric comparison (CO) subjects. (**B**) Targets of miRNA differentially expressed in schizophrenia subjects with severe cognitive deficits (CD) compared to schizophrenia subjects with spared cognition (CS). (**C**) Targets of miRNA differentially expressed in CD compared to CO subjects. Related to Table 2 and Fig. 3.

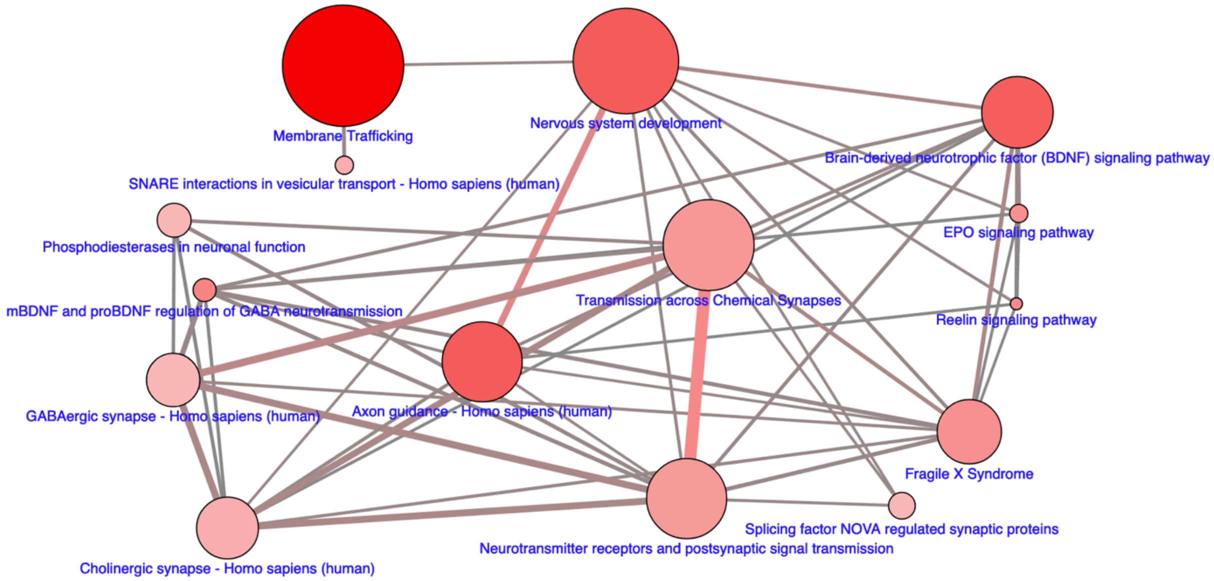

Figure S9 Cognitive deficit associated miRNA pathways. Original image from pathways analysis using Consensus Path Database, with edges filtered for minimum relative overlap 0.08. Related to Fig. 4

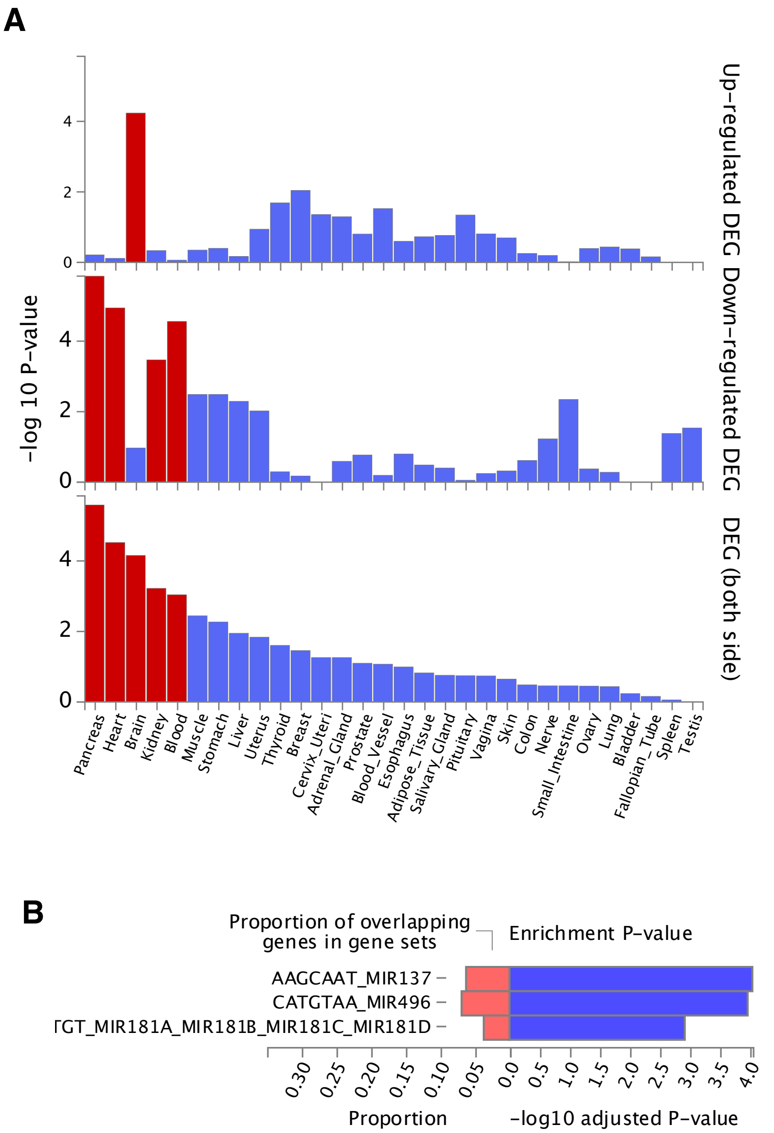

Fig. S10 Consistently dysregulated miR-1246 target genes. Predicted targets of hsa-miR-1246 were determined with TargetScan and filtered to include high confidence genes based on binding site efficacy; cumulative weighted context score less than -0.2. (**A**) Target genes are specifically enriched in the brain and (**B**) significantly overlap with miR-137 targets.

Table S1. Enrichment analysis of putative brain associated miRNA expressed in the full cohort. Enrichment analysis for the set of miRNA detected (105 miRNA) in the whole cohort (477 subjects) using all miRNA in the TAM2.0 database. Adjusted *P* values by Bonferroni and false discovery rate.

**Category**	**Term**	**Count**	***P***	**Bonferroni**	**FDR**	**miRNA**
Tissue specificity	Brain (cerebellum)	16	1.08*e*-16	6.56*e*-14	6.22*e*-15	miR-16-1, miR-16-2, miR-185, miR-19b-1, miR-19b-2, miR-93, miR-451a, miR-25, miR-223, miR-144, miR-15b, miR-19a, miR-486-1, miR-486-2, miR-20a, miR-17
Cluster	miR-17 cluster	5	3.71*e*-06	2.25*e*-03	4.56*e*-05	miR-17, miR-19a, miR-20a, miR-19b-1, miR-92a-1
Family	let-7 family	12	9.46*e*-16	5.74*e*-13	4.96*e*-14	let-7a-1, let-7a-2, let-7a-3, let-7b, let-7c, let-7d, let-7e, let-7f-1, let-7f-2, let-7g, let-7i, miR-98
Family	miR-29 family	4	1.14*e*-05	6.93*e*-03	1.27*e*-04	miR-29a, miR-29b-1, miR-29b-2, miR-29c

Table S2. Expression correlation for miR-17~92 cluster molecules. Spearman's correlation analysis of normalised counts per million indicates expression of hsa-miR-19a-3p and hsa-miR-19b-3p from serum extracellular vesicles enriched for neuronal origin have a positive monotonic association (Spearman's rho 0.70).

	miR-17-5p	**miR-19a-3p**	**miR-19b-3p**	miR-20a-5p	miR-92a-3p	miR-25-3p	miR-93-5p
miR-17-5p	1.00	0.36	0.38	0.36	0.28	0.05	0.42
**miR-19a-3p**	0.36	1.00	**0.70**	0.40	0.17	0.17	0.34
**miR-19b-3p**	0.38	**0.70**	1.00	0.41	0.14	0.15	0.33
miR-20a-5p	0.36	0.40	0.41	1.00	0.40	0.08	0.35
miR-92a-3p	0.28	0.17	0.14	0.40	1.00	0.17	0.38
miR-25-3p	0.05	0.17	0.15	0.08	0.17	1.00	0.25
miR-93-5p	0.42	0.34	0.33	0.35	0.38	0.25	1.00

Table S3. miRNA expression in subtypes of schizophrenia. Treatment resistant schizophrenia (TRS): taking clozapine at the time of assessment, n=42. Non-TRS: schizophrenia subjects not taking clozapine at the time of assessment, n=179. Non-psychiatric comparison subjects, n=256. Early onset schizophrenia (EOS): onset before 18 years of age, n=31. Non-EOS: onset at 18 years of age and onwards, n=190. FDR=false discovery rate adjusted *P* value.

mature miRNA name	log_2 fold change	FDR
Treatment resistant schizophrenia compared to non-treatment resistant schizophrenia (cases only, 196 miRNA)		
hsa-miR-1246	1.752	7.66E-08
hsa-miR-5100	2.074	1.19E-06
hsa-miR-7704	2.421	4.70E-06
hsa-miR-4521	1.989	5.13E-05
hsa-miR-10396a-5p	1.852	1.95E-02
hsa-miR-3178	1.270	3.24E-02
hsa-miR-3615	1.963	3.74E-02
hsa-miR-203a-3p	1.023	6.32E-02
hsa-miR-199a-5p	-1.286	6.32E-02
hsa-miR-1843	1.260	9.29E-02
Treatment resistant schizophrenia compared to non-treatment resistant schizophrenia plus comparison subjects (cases and comparison), unadjusted		
hsa-miR-1246	2.287	7.23E-17
hsa-miR-5100	2.332	1.40E-11
hsa-miR-7704	2.548	2.62E-09
hsa-miR-4521	1.934	2.73E-08
hsa-miR-203a-3p	1.411	9.96E-05
hsa-miR-3178	1.301	2.77E-03
hsa-miR-199a-5p	-1.255	1.30E-02
hsa-miR-590-5p	-1.326	2.72E-02
hsa-miR-146a-5p	-0.822	3.44E-02
Early onset schizophrenia (<18 years) compared to non-early onset schizophrenia (cases only, 105 miRNA)		
hsa-miR-3175	-2.480	9.35E-01
hsa-miR-223-3p	-0.570	9.35E-01
Differentially expressed miRNA in the context of positive symptoms		
hsa-let-7b-5p	-0.051	8.60E-01
hsa-miR-23b-3p	-0.069	8.60E-01
Differentially expressed miRNA in the context of negative symptoms		
hsa-miR-4485-5p	0.245	8.61E-01
hsa-miR-664a-3p	-0.384	8.61E-01
hsa-miR-10b-5p	-0.246	9.58E-01
hsa-miR-451a	0.189	9.58E-01
hsa-miR-142-3p	-0.160	9.58E-01

Table S4. Gene and disease ontologies enriched for genes targeted by miRNA that are dysregulated in schizophrenia subjects compared to comparison subjects. n=the number of annotated genes from the input list, %=n/total number of genes from the input list (total 715).

GO: Biological process					
**Ontology ID**	**Ontology name**	**n**	**%**	***P***	**Bonferroni**
GO:0050807	regulation of synapse organisation	26	3.6	1.690E-6	1.071E-2
GO:0050803	regulation of synapse structure or activity	26	3.6	4.086E-6	2.590E-2
GO: Cellular component					
GO:0099060	integral component of postsynaptic specialization membrane	14	2.0	1.563E-5	1.230E-2
GO:0099055	integral component of postsynaptic membrane	18	2.5	2.123E-5	1.671E-2
GO:0014069	postsynaptic density	33	4.6	2.304E-5	1.813E-2
GO:0032279	asymmetric synapse	33	4.6	2.304E-5	1.813E-2
GO:0098948	intrinsic component of postsynaptic specialization membrane	14	2.0	2.941E-5	2.314E-2
GO: Molecular function					
GO:1990837	sequence-specific double-stranded DNA binding	110	15.4	7.341E-10	9.286E-7
GO:0003690	double-stranded DNA binding	114	15.9	9.334E-10	1.181E-6
GO:0000976	transcription cis-regulatory region binding	107	15.0	1.339E-9	1.694E-6
GO:0001067	transcription regulatory region nucleic acid binding	107	15.0	1.582E-9	2.002E-6
GO:0003700	DNA-binding transcription factor activity	98	13.7	2.106E-9	2.664E-6

Table S5. Gene and disease ontologies enriched for genes targeted by miRNA that are dysregulated in schizophrenia subjects with cognitive deficit compared to comparison subjects. n=the number of annotated genes from the input list, %=n/total number of genes from the input list (total 6245).

GO: Biological process					
**Ontology ID**	**Ontology name**	**n**	**%**	***P***	**Bonferroni**
GO:0000902	cell morphogenesis	600	9.6	5.609E-27	7.616E-23
GO:0048666	neuron development	685	11.0	5.701E-26	7.740E-22
GO:0120039	plasma membrane bounded cell projection morphogenesis	424	6.8	3.023E-24	4.104E-20
GO:0031175	neuron projection development	586	9.4	4.609E-24	6.258E-20
GO:0048858	cell projection morphogenesis	426	6.8	5.684E-24	7.717E-20
GO: Cellular component					
GO:0045202	synapse	684	11.0	3.664E-25	6.076E-22
GO:0005794	Golgi apparatus	717	11.5	1.752E-21	2.904E-18
GO:0070161	anchoring junction	555	8.9	2.092E-16	3.468E-13
GO:0098794	postsynapse	345	5.5	1.735E-15	2.877E-12
GO:0000139	Golgi membrane	311	5.0	4.125E-15	6.839E-12
GO: Molecular function					
GO:0043565	sequence-specific DNA binding	747	12.0	1.662E-26	5.851E-23
GO:0003700	DNA-binding transcription factor activity	637	10.2	3.071E-25	1.081E-21
GO:0000981	DNA-binding transcription factor activity, RNA polymerase II-specific	615	9.8	8.420E-25	2.965E-21
GO:1990837	sequence-specific double-stranded DNA binding	697	11.2	1.528E-24	5.379E-21
GO:0001067	transcription regulatory region nucleic acid binding	676	10.8	3.887E-24	1.369E-20

Table S6. Gene and disease ontologies enriched for genes targeted by miRNA that are dysregulated in schizophrenia subjects with cognitive deficit compared to cognitively spared schizophrenia subjects. n=the number of annotated genes from the input list, %=n/total number of genes from the input list (total 2958).

GO: Biological process					
**Ontology ID**	**Ontology name**	**n**	**%**	***P***	**Bonferroni**
GO:0048699	generation of neurons	371	12.5	8.007E-15	8.467E-11
GO:0022008	neurogenesis	388	13.1	1.580E-13	1.671E-9
GO:0030182	neuron differentiation	335	11.3	5.378E-13	5.687E-9
GO:0000902	cell morphogenesis	254	8.6	9.027E-12	9.545E-8
GO:0048858	cell projection morphogenesis	181	6.1	1.765E-11	1.866E-7
GO: Cellular component					
GO:0045202	synapse	322	10.9	3.203E-12	4.109E-9
GO:0098791	Golgi apparatus subcompartment	203	6.9	1.822E-10	2.337E-7
GO:0030424	axon	187	6.3	2.632E-10	3.377E-7
GO:0005794	Golgi apparatus	332	11.2	5.207E-10	6.681E-7
GO:0000139	Golgi membrane	174	5.9	1.850E-9	2.373E-6
GO: Molecular function					
GO:0140110	transcription regulator activity	410	13.9	6.737E-14	1.623E-10
GO:0001067	transcription regulatory region nucleic acid binding	353	11.9	2.932E-13	7.062E-10
GO:0003700	DNA-binding transcription factor activity	318	10.8	1.285E-12	3.096E-9
GO:0000976	transcription cis-regulatory region binding	349	11.8	1.311E-12	3.159E-9
GO:1990837	sequence-specific double-stranded DNA binding	357	12.1	1.318E-12	3.174E-9

Table S7. Gene and disease ontologies enriched for genes targeted by miRNA that are dysregulated in subjects with treatment resistant schizophrenia compared to schizophrenia subjects not treatment resistant. n=the number of annotated genes from the input list, %=n/total number of genes from the input list (total 5911).

GO: Biological process					
**Ontology ID**	**Ontology name**	**n**	**%**	***P***	**Bonferroni**
GO:0048666	neuron development	654	11.1	1.353E-25	1.812E-21
GO:0000902	cell morphogenesis	557	9.4	2.244E-22	3.006E-18
GO: 0031175	neuron projection development	553	9.4	4.843E-22	6.487E-18
GO: 0048858	cell projection morphogenesis	401	6.8	1.122E-21	1.503E-17
GO: 0120039	plasma membrane bounded cell projection morphogenesis	398	6.7	1.332E-21	1.784E-17
GO: Cellular component					
GO:0045202	synapse	671	11.4	2.454E-29	4.030E-26
GO:0070161	anchoring junction	548	9.3	2.562E-20	4.208E-17
GO:0098794	postsynapse	346	5.9	8.868E-20	1.456E-16
GO:0043005	neuron projection	658	11.1	1.562E-18	2.565E-15
GO:0005794	Golgi apparatus	669	11.3	3.881E-18	6.373E-15
GO: Molecular function					
GO:0043565	sequence-specific DNA binding	710	12.0	2.678E-25	9.242E-22
GO:0000981	DNA-binding transcription factor activity, RNA polymerase II-specific	591	10.0	2.872E-25	9.913E-22
GO:0003700	DNA-binding transcription factor activity	610	10.3	3.440E-25	1.187E-21
GO:1990837	sequence-specific double-stranded DNA binding	662	11.2	2.543E-23	8.776E-20
GO:0001067	transcription regulatory region nucleic acid binding	643	10.9	3.737E-23	1.290E-19

Table S8. Gene and disease ontologies enriched for genes targeted by miRNA that are dysregulated in subjects with treatment resistant schizophrenia compared to not treatment resistant subjects, adjusted for case comparison status. n=the number of annotated genes from the input list, %=n/total number of genes from the input list (total 6079).

GO: Biological process					
**Ontology ID**	**Ontology name**	**n**	**%**	***P***	**Bonferroni**
GO:0048666	neuron development	674	11.1	5.484E-27	7.421E-23
GO:0000902	cell morphogenesis	575	9.5	8.055E-24	1.090E-19
GO: 0031175	neuron projection development	572	9.4	9.482E-24	1.283E-19
GO:0048858	cell projection morphogenesis	414	6.8	5.057E-23	6.843E-19
GO:0120039	plasma membrane bounded cell projection morphogenesis	411	6.8	5.695E-23	7.706E-19
GO: Cellular component					
GO:0045202	synapse	685	11.3	3.902E-29	6.443E-26
GO:0070161	anchoring junction	562	9.2	1.056E-20	1.744E-17
GO:0043005	neuron projection	675	11.1	7.329E-19	1.210E-15
GO:0098794	postsynapse	350	5.8	7.909E-19	1.306E-15
GO:0005794	Golgi apparatus	686	11.3	2.148E-18	3.546E-15
GO: Molecular function					
GO:0043565	sequence-specific DNA binding	728	12.0	8.958E-26	3.145E-22
GO:0003700	DNA-binding transcription factor activity	625	10.3	1.379E-25	4.840E-22
GO:0000981	DNA-binding transcription factor activity, RNA polymerase II-specific	605	10.0	1.537E-25	5.395E-22
GO:1990837	sequence-specific double-stranded DNA binding	679	11.2	8.337E-24	2.927E-20
GO:0001067	transcription regulatory region nucleic acid binding	659	10.8	1.612E-23	5.661E-20

Table S9 Gene set analysis. Multi-marker Analysis of GenoMic Annotation (MAGMA) (*84*) identifies significant association between genomic risk for schizophrenia and predicted target genes of dysregulated miRNA. SZ=schizophrenia, CO=non-psychiatric comparison, CD=cognitive deficit subtype schizophrenia, CS=cognitive spared subtype schizophrenia.

**SET**	**BETA**	**SE**	***P***	**CONFIGURATION**
CDvsCO	0.1283	0.023	1.81E-08	Conservative genic boundaries
CDvsCS	0.078	0.023	4.81E-04	Conservative genic boundaries
SZvsCO	0.086	0.037	9.36E-06	Conservative genic boundaries
CDvsCO	0.128	0.024	2.43E-08	Conservative genic boundaries (expression covariation)
CDvsCS	0.076	0.024	7.06E-04	Conservative genic boundaries (expression covariation)
SZvsCO	0.087	0.02	1.82E-05	Conservative genic boundaries (expression covariation)
CDvsCO	0.119	0.026	2.21E-06	Liberal genic boundaries
CDvsCS	0.096	0.026	1.12E-04	Liberal genic boundaries
SZvsCO	0.091	0.023	3.03E-05	Liberal genic boundaries

Auxiliary supplementary tables;

1. **Table S10**
    a. Supplementary_materials_TableS10.xlsx
    b. Title: CNS expression and previous association with schizophrenia for differentially expressed miRNA.
    c. Description: Manual annotation of differentially expressed miRNA with respect to human CNS expression and previous association with schizophrenia.
2. **Table S11**
    a. Supplementary_materials_TableS11.xlsx
    b. Title: Demographics and characteristics of individual subjects.
    c. Description: Detailed characteristics of subjects (n=477), including diagnosis, age of onset, antipsychotic medications and symptom scores.

REFERENCES AND NOTES

1. B. J. Goldie, M. D. Dun, M. Lin, N. D. Smith, N. M. Verrills, C. V. Dayas, M. J. Cairns, Activity-associated miRNA are packaged in Map1b-enriched exosomes released from depolarized neurons. *Nucleic Acids Res.* **42**, 9195–208 (2014).

2. K. L. Stark, B. Xu, A. Bagchi, W.-S. Lai, H. Liu, R. Hsu, X. Wan, P. Pavlidis, A. A. Mills, M. Karayiorgou, J. A. Gogos, Altered brain microRNA biogenesis contributes to phenotypic deficits in a 22q11-deletion mouse model. *Nat. Genet.* **40**, 751–760 (2008).

3. M. Shibata, H. Nakao, H. Kiyonari, T. Abe, S. Aizawa, MicroRNA-9 regulates neurogenesis in mouse telencephalon by targeting multiple transcription factors. *J. Neurosci.* **31**, 3407–3422 (2011).

4. I. Park, H. J. Kim, Y. Kim, H. S. Hwang, H. Kasai, J.-H. Kim, J. W. Park, Nanoscale imaging reveals miRNA-mediated control of functional states of dendritic spines. *Proc. Natl. Acad. Sci. U.S.A.* **116**, 9616–9621 (2019).

5. J. Gao, W.-Y. Wang, Y.-W. Mao, J. Gräff, J.-S. Guan, L. Pan, G. Mak, D. Kim, S. C. Su, L.-H. Tsai, A novel pathway regulates memory and plasticity via SIRT1 and miR-134. *Nature* **466**, 1105–1109 (2010).

6. K. F. Hansen, K. Sakamoto, G. A. Wayman, S. Impey, K. Obrietan, Transgenic miR132 alters neuronal spine density and impairs novel object recognition memory. *PLOS ONE* **5**, e15497 (2010).

7. N. J. Beveridge, E. Gardiner, A. P. Carroll, P. A. Tooney, M. J. Cairns, Schizophrenia is associated with an increase in cortical microRNA biogenesis. *Mol. Psychiatry* **15**, 1176–89 (2010).

8. D. M. Santarelli, N. J. Beveridge, P. A. Tooney, M. J. Cairns, Upregulation of dicer and microRNA expression in the dorsolateral prefrontal cortex Brodmann area 46 in schizophrenia. *Biol. Psychiatry* **69**, 180–7 (2011).

9. M. Chivet, C. Javalet, K. Laulagnier, B. Blot, F. J. Hemming, R. Sadoul, Exosomes secreted by cortical neurons upon glutamatergic synapse activation specifically interact with neurons. *J. Extracell. Vesicles* **3**, 24722 (2014).

10. M. Shi, C. Liu, T. J. Cook, K. M. Bullock, Y. Zhao, C. Ginghina, Y. Li, P. Aro, R. Dator, C. He, M. J. Hipp, C. P. Zabetian, E. R. Peskind, S. C. Hu, J. F. Quinn, D. R. Galasko, W. A. Banks, J. Zhang, Plasma exosomal α-synuclein is likely CNS-derived and increased in Parkinson's disease. *Acta Neuropathol.* **128**, 639–50 (2014).

11. M. S. Fiandaca, D. Kapogiannis, M. Mapstone, A. Boxer, E. Eitan, J. B. Schwartz, E. L. Abner, R. C. Petersen, H. J. Federoff, B. L. Miller, E. J. Goetzl, Identification of preclinical Alzheimer's disease by a profile of pathogenic proteins in neurally derived blood exosomes: A case-control study. *Alzheimers Dement.* **11**, 600–7.e1 (2015).

12. E. J. Goetzl, A. Boxer, J. B. Schwartz, E. L. Abner, R. C. Petersen, B. L. Miller, O. D. Carlson, M. Mustapic, D. Kapogiannis, Low neural exosomal levels of cellular survival factors in Alzheimer's disease. *Ann. Clin. Transl. Neurol.* **2**, 769–773 (2015).

13. D. Kapogiannis, A. Boxer, J. B. Schwartz, E. L. Abner, A. Biragyn, U. Masharani, L. Frassetto, R. C. Petersen, B. L. Miller, E. J. Goetzl, Dysfunctionally phosphorylated type 1 insulin receptor substrate in neural-derived blood exosomes of preclinical Alzheimer's disease. *FASEB J.* **29**, 589–96 (2015).

14. M. Mustapic, E. Eitan, J. K. J. Werner, S. T. Berkowitz, M. P. Lazaropoulos, J. Tran, E. J. Goetzl, D. Kapogiannis, Plasma extracellular vesicles enriched for neuronal origin: A potential window into brain pathologic processes. *Front. Neurosci.* **11,** 278 (2017).

15. M. Qu, Q. Lin, L. Huang, Y. Fu, L. Wang, S. He, Y. Fu, S. Yang, Z. Zhang, L. Zhang, X. Sun, Dopamine-loaded blood exosomes targeted to brain for better treatment of Parkinson's disease. *J. Control. Release* **287**, 156–166 (2018).

16. D. J. Gibbings, C. Ciaudo, M. Erhardt, O. Voinnet, Multivesicular bodies associate with components of miRNA effector complexes and modulate miRNA activity. *Nat. Cell Biol.* **11**, 1143–1149 (2009).

17. Y. S. Lee, S. Pressman, A. P. Andress, K. Kim, J. L. White, J. J. Cassidy, X. Li, K. Lubell, D. H. Lim, I. S. Cho, K. Nakahara, J. B. Preall, P. Bellare, E. J. Sontheimer, R. W. Carthew, Silencing by small RNAs is linked to endosomal trafficking. *Nat. Cell Biol.* **11**, 1150–1156 (2009).

18. S. Ghosh, M. Bose, A. Ray, S. N. Bhattacharyya, Polysome arrest restricts miRNA turnover by preventing exosomal export of miRNA in growth-retarded mammalian cells. *Mol. Biol. Cell* **26**, 1072–83 (2015).

19. S. Saeedi, C. Nagy, P. Ibrahim, J.-F. Théroux, M. Wakid, L. M. Fiori, J. Yang, S. Rotzinger, J. A. Foster, N. Mechawar, S. H. Kennedy, G. Turecki, Neuron-derived extracellular vesicles enriched from plasma show altered size and miRNA cargo as a function of antidepressant drug response. *Mol. Psychiatry* **26,** 7417–7424 (2021).

20. M. Norman, D. Ter-Ovanesyan, W. Trieu, R. Lazarovits, E. J. K. Kowal, J. H. Lee, A. S. Chen-Plotkin, A. Regev, G. M. Church, D. R. Walt, L1CAM is not associated with extracellular vesicles in human cerebrospinal fluid or plasma. *Nat. Methods* **18**, 631–634 (2021).

21. L. J. Vella, B. J. Scicluna, L. Cheng, E. G. Bawden, C. L. Masters, C.-S. Ang, N. Willamson, C. McLean, K. J. Barnham, A. F. Hill, A rigorous method to enrich for exosomes from brain tissue. *J. Extracell. Vesicles* **6**, 1348885 (2017).

22. S. L. Tanner, R. Franzen, H. Jaffe, R. H. Quarles, Evidence for expression of some microtubule-associated protein 1B in neurons as a plasma membrane glycoprotein. *J. Neurochem.* **75**, 553–562 (2000).

23. C. A. de Leeuw, S. Stringer, I. A. Dekkers, T. Heskes, D. Posthuma, Conditional and interaction gene-set analysis reveals novel functional pathways for blood pressure. *Nat. Commun.* **9**, 3768 (2018).

24. N. J. Beveridge, P. A. Tooney, A. P. Carroll, N. Tran, M. J. Cairns, Down-regulation of miR-17 family expression in response to retinoic acid induced neuronal differentiation. *Cell. Signal.* **21**, 1837–1845 (2009).

25. H. Xin, M. Katakowski, F. Wang, J. Y. Qian, X. S. Liu, M. M. Ali, B. Buller, Z. G. Zhang, M. Chopp, MicroRNA cluster miR-17-92 cluster in exosomes enhance neuroplasticity and functional recovery after stroke in rats. *Stroke* **48**, 747–753 (2017).

26. J. Han, H. J. Kim, S. T. Schafer, A. Paquola, G. D. Clemenson, T. Toda, J. Oh, A. R. Pankonin, B. S. Lee, S. T. Johnston, A. Sarkar, A. M. Denli, F. H. Gage, Functional implications of miR-19 in the migration of newborn neurons in the adult brain. *Neuron* **91**, 79–89 (2016).

27. G. Bieri, A. B. Schroer, S. A. Villeda, Blood-to-brain communication in aging and rejuvenation. *Nat. Neurosci.* **26**, 379–393 (2023).

28. P. Landgraf, M. Rusu, R. Sheridan, A. Sewer, N. Iovino, A. Aravin, S. Pfeffer, A. Rice, A. O. Kamphorst, M. Landthaler, C. Lin, N. D. Socci, L. Hermida, V. Fulci, S. Chiaretti, R. Foà, J. Schliwka, U. Fuchs, A. Novosel, R.-U. Müller, B. Schermer, U. Bissels, J. Inman, Q. Phan, M. Chien, D. B. Weir, R. Choksi, G. De Vita, D. Frezzetti, H.-I. Trompeter, V. Hornung, G. Teng, G. Hartmann, M. Palkovits, R. Di Lauro, P. Wernet, G. Macino, C. E. Rogler, J. W. Nagle, J. Ju, F. N. Papavasiliou, T. Benzing, P. Lichter, W. Tam, M. J. Brownstein, A. Bosio, A. Borkhardt, J. J. Russo, C. Sander, M. Zavolan, T. Tuschl, A mammalian microRNA expression atlas based on small rna library sequencing. *Cell* **129**, 1401–1414 (2007).

29. J. T. G. Pena, C. Sohn-Lee, S. H. Rouhanifard, J. Ludwig, M. Hafner, A. Mihailovic, C. Lim, D. Holoch, P. Berninger, M. Zavolan, T. Tuschl, miRNA in situ hybridization in formaldehyde and EDC–fixed tissues. *Nat. Methods* **6**, 139–141 (2009).

30. B. Panwar, G. S. Omenn, Y. Guan, miRmine: A database of human miRNA expression profiles. *Bioinformatics* **33**, 1554–1560 (2017).

31. E. Hanson, K. Healey, D. Wolf, C. Kohler, Assessment of pharmacotherapy for negative symptoms of schizophrenia. *Curr. Psychiatry Rep.* **12**, 563–571 (2010).

32. P. D. Harvey, R. K. Heaton, W. T. Carpenter, M. F. Green, J. M. Gold, M. Schoenbaum, Functional impairment in people with schizophrenia: Focus on employability and eligibility for disability compensation. *Schizophr. Res.* **140**, 1–8 (2012).

33. M. J. Green, M. J. Cairns, J. Wu, M. Dragovic, A. Jablensky, P. A. Tooney, R. J. Scott, V. J. Carr, Genome-wide supported variant MIR137 and severe negative symptoms predict membership of an impaired cognitive subtype of schizophrenia. *Mol. Psychiatry* **18**, 774–780 (2013).

34. F. Iasevoli, S. Giordano, R. Balletta, G. Latte, M. V. Formato, E. Prinzivalli, D. De Berardis, C. Tomasetti, A. de Bartolomeis, Treatment resistant schizophrenia is associated with the worst community functioning among severely-ill highly-disabling psychiatric conditions and is the most relevant predictor of poorer achievements in functional milestones. *Prog. Neuropsychopharmacol. Biol. Psychiatry* **65**, 34–48 (2016).

35. S. Ripke, C. O'Dushlaine, K. Chambert, J. L. Moran, A. K. Kahler, S. Akterin, S. E. Bergen, A. L. Collins, J. J. Crowley, M. Fromer, Y. Kim, S. H. Lee, P. K. Magnusson, N. Sanchez, E. A. Stahl, S. Williams, N. R. Wray, K. Xia, F. Bettella, A. D. Borglum, B. K. Bulik-Sullivan, P. Cormican, N. Craddock, C. de Leeuw, N. Durmishi, M. Gill, V. Golimbet, M. L. Hamshere, P. Holmans, D. M. Hougaard, K. S. Kendler, K. Lin, D. W. Morris, O. Mors, P. B. Mortensen, B. M. Neale, F. A. O'Neill, M. J. Owen, M. P. Milovancevic, D. Posthuma, J. Powell, A. L. Richards, B. P. Riley, D. Ruderfer, D. Rujescu, E. Sigurdsson, T. Silagadze, A. B. Smit, H. Stefansson, S. Steinberg, J. Suvisaari, S. Tosato, M. Verhage, J. T. Walters, D. F. Levinson, P. V. Gejman, K. S. Kendler, C. Laurent, B. J. Mowry, M. C. O'Donovan, M. J. Owen, A. E. Pulver, B. P. Riley, S. G. Schwab, D. B. Wildenauer, F. Dudbridge, P. Holmans, J. Shi, M. Albus, M. Alexander, D. Campion, D. Cohen, D. Dikeos, J. Duan, P. Eichhammer, S. Godard, M. Hansen, F. B. Lerer, K. Y. Liang, W. Maier, J. Mallet, D. A. Nertney, G. Nestadt, N. Norton, F. A. O'Neill, G. N. Papadimitriou, R. Ribble, A. R. Sanders, J. M. Silverman, D. Walsh, N. M. Williams, B. Wormley, M. J. Arranz, S. Bakker, S. Bender, E. Bramon, D. Collier, B. Crespo-Facorro, J. Hall, C. Iyegbe, A. Jablensky, R. S. Kahn, L. Kalaydjieva, S. Lawrie, C. M. Lewis, K. Lin, D. H. Linszen, I. Mata, A. McIntosh, R. M. Murray, R. A. Ophoff, J. Powell, D. Rujescu, J. Van Os, M. Walshe, M. Weisbrod, D. Wiersma, P. Donnelly, I. Barroso, J. M. Blackwell, E. Bramon, M. A. Brown, J. P. Casas, A. P. Corvin, P. Deloukas, A. Duncanson, J. Jankowski, H. S. Markus, C. G. Mathew, C. N. Palmer, R. Plomin, A. Rautanen, S. J. Sawcer, R. C. Trembath, A. C. Viswanathan, N. W. Wood, C. C. Spencer, G. Band, C. Bellenguez, C. Freeman, G. Hellenthal, E. Giannoulatou, M. Pirinen, R. D. Pearson, A. Strange, Z. Su, D. Vukcevic, P. Donnelly, C. Langford, S. E. Hunt, S. Edkins, R. Gwilliam, H. Blackburn, S. J. Bumpstead, S. Dronov, M. Gillman, E. Gray, N. Hammond, A. Jayakumar, O. T. McCann, J. Liddle,

S. C. Potter, R. Ravindrarajah, M. Ricketts, A. Tashakkori-Ghanbaria, M. J. Waller, P. Weston, S. Widaa, P. Whittaker, I. Barroso, P. Deloukas, C. G. Mathew, J. M. Blackwell, M. A. Brown, A. P. Corvin, M. I. McCarthy, C. C. Spencer, E. Bramon, A. P. Corvin, M. C. O'Donovan, K. Stefansson, E. Scolnick, S. Purcell, S. A. McCarroll, P. Sklar, C. M. Hultman, P. F. Sullivan, Genome-wide association analysis identifies 13 new risk loci for schizophrenia. *Nat. Genet.* **45**, 1150–1159 (2013).

36. E. Kwon, W. Wang, L. H. Tsai, Validation of schizophrenia-associated genes CSMD1, C10orf26, CACNA1C and TCF4 as miR-137 targets. *Mol. Psychiatry* **18**, 11–12 (2013).

37. C. Wright, V. D. Calhoun, S. Ehrlich, L. Wang, J. A. Turner, N. I. Perrone-Bizzozero, Meta gene set enrichment analyses link miR-137-regulated pathways with schizophrenia risk. *Front. Genet.* **6,** 147 (2015).

38. K. T. Thomas, C. Gross, G. J. Bassell, microRNAs sculpt neuronal communication in a tight balance that is lost in neurological disease. *Front. Mol. Neurosci.* **11**, 455 (2018).

39. P. M. McKeever, R. Schneider, F. Taghdiri, A. Weichert, N. Multani, R. A. Brown, A. L. Boxer, A. Karydas, B. Miller, J. Robertson, M. C. Tartaglia, MicroRNA expression levels are altered in the cerebrospinal fluid of patients with young-onset Alzheimer's disease. *Mol. Neurobiol.* **55**, 8826–8841 (2018).

40. J. A. Gallego, M. L. Gordon, K. Claycomb, M. Bhatt, T. Lencz, A. K. Malhotra, In vivo microRNA detection and quantitation in cerebrospinal fluid. *J. Mol. Neurosci.* **47**, 243–8 (2012).

41. J. Gallego, E. Alsop, T. Lencz, K. Van Keuren-Jensen, A. Malhotra, F10. Differential expression of microRNAs in cerebrospinal fluid and plasma samples in schizophrenia. *Schizophr. Bull.* **44**, S221–S222 (2018).

42. M. G. Banigan, P. F. Kao, J. A. Kozubek, A. R. Winslow, J. Medina, J. Costa, A. Schmitt, A. Schneider, H. Cabral, O. Cagsal-Getkin, C. R. Vanderburg, I. Delalle, Differential expression of exosomal microRNAs in prefrontal cortices of schizophrenia and bipolar disorder patients. *PLOS ONE* **8**, e48814 (2013).

43. K. E. Burns, K. D. Deane-Alder, B. L. Bellissima, M. D. Tingle, Circulating microRNA as biomarkers of clozapine-induced cardiotoxicity. *Biomarkers* **25**, 76–85 (2020).

44. D. O. Perkins, C. D. Jeffries, L. F. Jarskog, J. M. Thomson, K. Woods, M. A. Newman, J. S. Parker, J. Jin, S. M. Hammond, microRNA expression in the prefrontal cortex of individuals with schizophrenia and schizoaffective disorder. *Genome Biol.* **8**, R27 (2007).

45. D. M. Santarelli, B. Liu, C. E. Duncan, N. J. Beveridge, P. A. Tooney, P. R. Schofield, M. J. Cairns, Gene-microRNA interactions associated with antipsychotic mechanisms and the metabolic side effects of olanzapine. *Psychopharmacology* **227**, 67–78 (2013).

46. T. S. Wang, Y. C. Chen, F. M. Tsai, Y. Y. Chang, Y. H. Chen, C. Y. Kuo, M. L. Chen, MicroRNA expression profiles in C6 glioblastoma cell line treated with antipsychotics. *Indian J. Pharm. Sci.* **82**, 409–419 (2020).

47. C. J. Bachmann, L. Aagaard, M. Bernardo, L. Brandt, M. Cartabia, A. Clavenna, A. Coma Fusté, K. Furu, K. Garuoliené, F. Hoffmann, S. Hollingworth, K. F. Huybrechts, L. J. Kalverdijk, K. Kawakami, H. Kieler, T. Kinoshita, S. C. López, J. E. Machado-Alba, M. E. Machado-Duque, M. Mahesri, P. S. Nishtala, D. Piovani, J. Reutfors, L. K. Saastamoinen, I. Sato, C. C. M. Schuiling-Veninga, Y.-C. Shyu, D. Siskind, S. Skurtveit, H. Verdoux, L.-J. Wang, C. Zara Yahni, H. Zoëga, D. Taylor, International trends in clozapine use: A study in 17 countries. *Acta Psychiatr. Scand.* **136**, 37–51 (2017).

48. S. Guo, J. Liu, W. Li, Y. Yang, L. Lv, X. Xiao, M. Li, F. Guan, X.-J. Luo, Genome wide association study identifies four loci for early onset schizophrenia. *Transl. Psychiatry* **11**, 248 (2021).

49. J. K. Forsyth, R. F. Asarnow, Genetics of childhood-onset schizophrenia 2019 update. *Child Adolesc. Psychiatr. Clin. N. Am.* **29**, 157–170 (2020).

50. D. Zhao, M. Lin, J. Chen, E. Pedrosa, A. Hrabovsky, H. M. Fourcade, D. Zheng, H. M. Lachman, MicroRNA profiling of neurons generated using induced pluripotent stem cells derived from patients with schizophrenia and schizoaffective disorder, and 22q11.2 Del. *PLOS ONE* **10**, e0132387 (2015).

51. Z. Zhao, S. Jinde, S. Koike, M. Tada, Y. Satomura, A. Yoshikawa, Y. Nishimura, R. Takizawa, A. Kinoshita, E. Sakakibara, H. Sakurada, M. Yamagishi, F. Nishimura, A. Inai, M. Nishioka, Y. Eriguchi, T. Araki, A. Takaya, C. Kan, M. Umeda, A. Shimazu, H. Hashimoto, M. Bundo, K. Iwamoto, C. Kakiuchi, K. Kasai, Altered expression of microRNA-223 in the plasma of patients with first-episode schizophrenia and its possible relation to neuronal migration-related genes. *Transl. Psychiatry* **9**, 289 (2019).

52. S. K. Amoah, B. A. Rodriguez, C. N. Logothetis, P. Chander, C. M. Sellgren, J. P. Weick, S. D. Sheridan, L. L. Jantzie, M. J. Webster, N. Mellios, Exosomal secretion of a psychosis-altered miRNA that regulates glutamate receptor expression is affected by antipsychotics. *Neuropsychopharmacology* **45,** 656–665 (2020).

53. K. S. Kendler, M. McGuire, A. M. Gruenberg, D. Walsh, Clinical heterogeneity in schizophrenia and the pattern of psychopathology in relatives: Results from an epidemiologically based family study. *Acta Psychiatr. Scand.* **89**, 294–300 (1994).

54. N. Andreasen, Symptoms, signs, and diagnosis of schizophrenia. *Lancet* **346**, 477–481 (1995).

55. C. Y. Lai, S. L. Yu, M. H. Hsieh, C. H. Chen, H. Y. Chen, C. C. Wen, Y. H. Huang, P. C. Hsiao, C. K. Hsiao, C. M. Liu, P. C. Yang, H. G. Hwu, W. J. Chen, MicroRNA expression aberration as potential peripheral blood biomarkers for schizophrenia. *PLOS ONE* **6**, e21635 (2011).

56. H. T. Song, X. Y. Sun, L. Zhang, L. Zhao, Z. M. Guo, H. M. Fan, A. F. Zhong, W. Niu, Y. H. Dai, L. Y. Zhang, Z. Shi, X. P. Liu, J. Lu, A preliminary analysis of association between the down-regulation of microRNA-181b expression and symptomatology improvement in schizophrenia patients before and after antipsychotic treatment. *J. Psychiatr. Res.* **54**, 134–40 (2014).

57. Y. Du, Y. Yu, Y. Hu, X.-W. Li, Z.-X. Wei, R.-Y. Pan, X.-S. Li, G.-E. Zheng, X.-Y. Qin, Q.-S. Liu, Y. Cheng, Genome-wide, integrative analysis implicates exosome-derived microrna dysregulation in schizophrenia. *Schizophr. Bull.* **45**, 1257–1266 (2019).

58. B. Schuler, J. Vogel, B. Grenacher, R. A. Jacobs, M. Arras, M. Gassmann, Acute and chronic elevation of erythropoietin in the brain improves exercise performance in mice without inducing erythropoiesis. *FASEB J.* **26**, 3884–3890 (2012).

59. P. T. Tsai, J. J. Ohab, N. Kertesz, M. Groszer, C. Matter, J. Gao, X. Liu, H. Wu, S. T. Carmichael, A critical role of erythropoietin receptor in neurogenesis and post-stroke recovery. *J. Neurosci.* **26**, 1269–1274 (2006).

60. D. Wakhloo, F. Scharkowski, Y. Curto, U. Javed Butt, V. Bansal, A. A. Steixner-Kumar, L. Wüstefeld, A. Rajput, S. Arinrad, M. R. Zillmann, A. Seelbach, I. Hassouna, K. Schneider, A. Qadir Ibrahim, H. B. Werner, H. Martens, K. Miskowiak, S. M. Wojcik, S. Bonn, J. Nacher, K.-A. Nave, H. Ehrenreich, Functional hypoxia drives neuroplasticity and neurogenesis via brain erythropoietin. *Nat. Commun.* **11**, 1313 (2020).

61. U. J. Butt, A. A. Steixner-Kumar, C. Depp, T. Sun, I. Hassouna, L. Wüstefeld, S. Arinrad, M. R. Zillmann, N. Schopf, L. Fernandez Garcia-Agudo, L. Mohrmann, U. Bode, A. Ronnenberg, M. Hindermann, S. Goebbels, S. Bonn, D. M. Katschinski, K. W. Miskowiak, K.-A. Nave, H. Ehrenreich, Hippocampal neurons respond to brain activity with functional hypoxia. *Mol. Psychiatry*, **26** (2021), 1790, 1807.

62. H. Ehrenreich, L. F. Garcia-Agudo, A. A. Steixner-Kumar, J. B. H. Wilke, U. J. Butt, Introducing the brain erythropoietin circle to explain adaptive brain hardware upgrade and improved performance. *Mol. Psychiatry* **27**, 2372–2379 (2022).

63. X.-B. Li, W. Zheng, Y.-P. Ning, D.-B. Cai, X.-H. Yang, G. S. Ungvari, C. H. Ng, C.-Y. Wang, Y.-T. Xiang, Erythropoietin for cognitive deficits associated with schizophrenia, bipolar disorder, and major depression: A systematic review. *Pharmacopsychiatry* **51**, 100–104 (2018).

64. C. Sauder, L. A. Allen, E. Baker, A. C. Miller, S. M. Paul, S. K. Brannan, Effectiveness of KarXT (xanomeline-trospium) for cognitive impairment in schizophrenia: Post hoc analyses from a randomised, double-blind, placebo-controlled phase 2 study. *Transl. Psychiatry* **12**, 491 (2022).

65. P. Maruff, E. Thomas, L. Cysique, B. Brew, A. Collie, P. Snyder, R. H. Pietrzak, Validity of the CogState brief battery: Relationship to standardized tests and sensitivity to cognitive impairment in mild traumatic brain injury, schizophrenia, and AIDS dementia complex. *Arch. Clin. Neuropsychol.* **24**, 165–178 (2009).

66. N. R. Livingston, P. C. Hawkins, J. Gilleen, R. Ye, L. Valdearenas, S. S. Shergill, M. A. Mehta, Preliminary evidence for the phosphodiesterase type-4 inhibitor, roflumilast, in ameliorating cognitive flexibility deficits in patients with schizophrenia. *J. Psychopharmacol.* **35**, 1099–1110 (2021).

67. X. Chen, M. Rosbash, MicroRNA-92a is a circadian modulator of neuronal excitability in Drosophila. *Nat. Commun.* **8**, 14707 (2017).

68. L. Zhou, C. Miller, L. J. Miraglia, A. Romero, L. S. Mure, S. Panda, S. A. Kay, A genome-wide microRNA screen identifies the microRNA-183/96/182 cluster as a modulator of circadian rhythms. *Proc. Natl. Acad. Sci. U.S.A.* **118**, e2020454118 (2021).

69. C. Loughland, D. Draganic, K. McCabe, J. Richards, A. Nasir, J. Allen, S. Catts, A. Jablensky, F. Henskens, P. Michie, B. Mowry, C. Pantelis, U. Schall, R. Scott, P. Tooney, V. Carr, Australian Schizophrenia Research Bank: A database of comprehensive clinical, endophenotypic and genetic data for aetiological studies of schizophrenia. *Aust. N. Z. J. Psychiatry* **44**, 1029–1035 (2010).

70. D. J. Castle, A. Jablensky, J. J. McGrath, V. Carr, V. Morgan, A. Waterreus, G. Valuri, H. Stain, P. McGuffin, A. Farmer, The diagnostic interview for psychoses (DIP): Development, reliability and applications. *Psychol. Med.* **36**, 69–80 (2006).

71. D. Hasin, M. L. Hatzenbuehler, K. Keyes, E. Ogburn, Substance Use Disorders: Diagnostic and Statistical Manual of Mental Disorders, fourth edition (DSM-IV) and International Classification of Diseases, tenth edition (ICD-10). *Addiction* **101**, 59–75 (2006).

72. D. Wechsler, *Wechsler Test of Adult Reading: WTAR* (Harcourt Assessment, San Antonio, TX, 2001).

73. D. Wechsler, *Wechsler Abbreviated Scale of Intelligence: WASI* (Harcourt Assessment, San Antonio, TX, 1999).

74. C. Randolph, *RBANS Manual-Repeatable Battery for the Assessment of Neuropsychological Status* (Harcourt Brace & Company, San Antonio, TX, 1998).

75. D. Wechsler, *Wechsler Adult Intelligence Scale -3rd Edition (WAIS-III)* (Harcourt Assessment, San Antonio, TX, 1997).

76. O. Spreen, A. Benton, *Neurosensory Center Comprehensive Examination for Aphasia (NCCEA)* (UVic press, Victoria, Canada, 1969).

77. J. Q. Wu, M. Green, E. J. Gardiner, P. A. Tooney, R. J. Scott, V. Carr, M. J. Cairns, Altered neural signaling and immune pathways in peripheral blood mononuclear cells of schizophrenia patients with cognitive impairment: a transcriptome analysis. *Brain Behav. Immun.* **53,** 194–206 (2015).

78. N. Andreasen, *The Scale for the Assessment of Negative Symptoms (SANS)* (IOWA press, Iowa City, 1983).

79. B. J. Tauro, D. W. Greening, R. A. Mathias, H. Ji, S. Mathivanan, A. M. Scott, R. J. Simpson, Comparison of ultracentrifugation, density gradient separation, and immunoaffinity capture methods for isolating human colon cancer cell line LIM1863-derived exosomes. *Methods* **56**, 293–304 (2012).

80. D. D. Taylor, S. Shah, Methods of isolating extracellular vesicles impact down-stream analyses of their cargoes. *Methods* **87**, 3–10 (2015).

81. D. J. McCarthy, Y. Chen, G. K. Smyth, Differential expression analysis of multifactor RNA-seq experiments with respect to biological variation. *Nucleic Acids Res.* **40**, 4288–4297 (2012).

82. V. Agarwal, G. W. Bell, J.-W. Nam, D. P. Bartel, Predicting effective microRNA target sites in mammalian mRNAs. *Elife* **4**, e05005 (2015).

83. V. Trubetskoy, A. F. Pardiñas, T. Qi, G. Panagiotaropoulou, S. Awasthi, T. B. Bigdeli, J. Bryois, C.-Y. Chen, C. A. Dennison, L. S. Hall, M. Lam, K. Watanabe, O. Frei, T. Ge, J. C. Harwood, F. Koopmans, S. Magnusson, A. L. Richards, J. Sidorenko, Y. Wu, J. Zeng, J. Grove, M. Kim, Z. Li, G. Voloudakis, W. Zhang, M. Adams, I. Agartz, E. G. Atkinson, E. Agerbo, M. Al Eissa, *M. Albus*, M. Alexander, B. Z. Alizadeh, K. Alptekin, T. D. Als, F. Amin, V. Arolt, M. Arrojo, L. Athanasiu, M. H. Azevedo, S. A. Bacanu, N. J. Bass, M. Begemann, R. A. Belliveau, J. Bene, B. Benyamin, S. E. Bergen, G. Blasi, J. Bobes, S. Bonassi, A. Braun, R. A. Bressan, E. J. Bromet, R. Bruggeman, P. F. Buckley, R. L. Buckner, J. Bybjerg-Grauholm, W. Cahn, M. J. Cairns, M. E. Calkins, V. J. Carr, D. Castle, S. V. Catts, K. D. Chambert, R. C. K. Chan, B. Chaumette, W. Cheng, E. F. C. Cheung, S. A. Chong, D. Cohen, A. Consoli, Q. Cordeiro, J. Costas, C. Curtis, M. Davidson, K. L. Davis, L. de Haan, F. Degenhardt, L. E. DeLisi, D. Demontis, F. Dickerson, D. Dikeos, T. Dinan, S. Djurovic, J. Duan, G. Ducci, F. Dudbridge, J. G. Eriksson, L. Fañanás, S. V. Faraone, A. Fiorentino, A. Forstner, J. Frank, N. B. Freimer, M. Fromer, A. Frustaci, A. Gadelha, G. Genovese, E. S. Gershon, M. Giannitelli, I. Giegling, P. Giusti-Rodríguez, S. Godard, J. I. Goldstein, J. González Peñas, A. González-Pinto, S. Gopal, J. Gratten, M. F. Green, T. A. Greenwood, O. Guillin, S. Gülöksüz, R. E. Gur, R. C. Gur, B. Gutiérrez, E. Hahn, H. Hakonarson, V. Haroutunian, A. M. Hartmann, C. Harvey, C. Hayward, F. A. Henskens, S. Herms, P. Hoffmann, D. P. Howrigan, M. Ikeda, C. Iyegbe, I. Joa, A. Julià, A. K. Kähler, T. Kam-Thong, Y. Kamatani, S. Karachanak-Yankova, O. Kebir, M. C. Keller, B. J. Kelly, A. Khrunin, S.-W. Kim, J. Klovins, N. Kondratiev, B. Konte, J. Kraft, M. Kubo, V. Kučinskas, Z. A. Kučinskiene, A. Kusumawardhani, H. Kuzelova-Ptackova, S. Landi, L. C. Lazzeroni, P. H. Lee, S. E. Legge, D. S. Lehrer, R. Lencer, B. Lerer, M. Li, J. Lieberman, G. A. Light, S. Limborska, C.-M. Liu, J. Lönnqvist, C. M. Loughland, J. Lubinski, J. J. Luykx, A. Lynham, M. Macek, A. Mackinnon, P. K. E. Magnusson, B. S. Maher, W. Maier, D. Malaspina, J. Mallet, S. R. Marder, S. Marsal, A. R. Martin, L. Martorell, M. Mattheisen, R. W. McCarley, C. McDonald, J. J. McGrath, H. Medeiros, S. Meier, B. Melegh, I. Melle, R. I. Mesholam-Gately, A. Metspalu, P. T. Michie, L. Milani, V. Milanova, M. Mitjans, E. Molden, E. Molina, M. D. Molto, V. Mondelli, C. Moreno, C. P. Morley, G. Muntané, K. C. Murphy, I. Myin-Germeys, I. Nenadić, G. Nestadt, L. Nikitina-Zake, C. Noto, K. H. Nuechterlein, N. L. O’Brien, F. A. O’Neill, S.-Y. Oh, A. Olincy, V. K. Ota, C. Pantelis, G. N. Papadimitriou, M. Parellada, T. Paunio, R. Pellegrino, S. Periyasamy, D. O. Perkins, B. Pfuhlmann, O. Pietiläinen, J. Pimm, D. Porteous, J. Powell, D. Quattrone, D. Quested, A. D. Radant, A. Rampino, M. H. Rapaport, A. Rautanen, A. Reichenberg,

C. Roe, J. L. Roffman, J. Roth, M. Rothermundt, B. P. F. Rutten, S. Saker-Delye, V. Salomaa, J. Sanjuan, M. L. Santoro, A. Savitz, U. Schall, R. J. Scott, L. J. Seidman, S. I. Sharp, J. Shi, L. J. Siever, E. Sigurdsson, K. Sim, N. Skarabis, P. Slominsky, H.-C. So, J. L. Sobell, E. Söderman, H. J. Stain, N. E. Steen, A. A. Steixner-Kumar, E. Stögmann, W. S. Stone, R. E. Straub, F. Streit, E. Strengman, T. S. Stroup, M. Subramaniam, C. A. Sugar, J. Suvisaari, D. M. Svrakic, N. R. Swerdlow, J. P. Szatkiewicz, T. M. T. Ta, A. Takahashi, C. Terao, F. Thibaut, D. Toncheva, P. A. Tooney, S. Torretta, S. Tosato, G. B. Tura, B. I. Turetsky, A. Üçok, A. Vaaler, T. van Amelsvoort, R. van Winkel, J. Veijola, J. Waddington, H. Walter, A. Waterreus, B. T. Webb, M. Weiser, N. M. Williams, S. H. Witt, B. K. Wormley, J. Q. Wu, Z. Xu, R. Yolken, C. C. Zai, W. Zhou, F. Zhu, F. Zimprich, E. C. Atbaşoğlu, M. Ayub, C. Benner, A. Bertolino, D. W. Black, N. J. Bray, G. Breen, N. G. Buccola, W. F. Byerley, W. J. Chen, C. R. Cloninger, B. Crespo-Facorro, G. Donohoe, R. Freedman, C. Galletly, M. J. Gandal, M. Gennarelli, D. M. Hougaard, H.-G. Hwu, A. V. Jablensky, S. A. McCarroll, J. L. Moran, O. Mors, P. B. Mortensen, B. Müller-Myhsok, A. L. Neil, M. Nordentoft, M. T. Pato, T. L. Petryshen, M. Pirinen, A. E. Pulver, T. G. Schulze, J. M. Silverman, J. W. Smoller, E. A. Stahl, D. W. Tsuang, E. Vilella, S.-H. Wang, S. Xu, Indonesia Schizophrenia Consortium, PsychENCODE, Psychosis Endophenotypes International Consortium, SynGO Consortium, R. Adolfsson, C. Arango, B. T. Baune, S. I. Belangero, A. D. Børglum, D. Braff, E. Bramon, J. D. Buxbaum, D. Campion, J. A. Cervilla, S. Cichon, D. A. Collier, A. Corvin, D. Curtis, M. D. Forti, E. Domenici, H. Ehrenreich, V. Escott-Price, T. Esko, A. H. Fanous, A. Gareeva, M. Gawlik, P. V. Gejman, M. Gill, S. J. Glatt, V. Golimbet, K. S. Hong, C. M. Hultman, S. E. Hyman, N. Iwata, E. G. Jönsson, R. S. Kahn, J. L. Kennedy, E. Khusnutdinova, G. Kirov, J. A. Knowles, M.-O. Krebs, C. Laurent-Levinson, J. Lee, T. Lencz, D. F. Levinson, Q. S. Li, J. Liu, A. K. Malhotra, D. Malhotra, A. McIntosh, A. McQuillin, P. R. Menezes, V. A. Morgan, D. W. Morris, B. J. Mowry, R. M. Murray, V. Nimgaonkar, M. M. Nöthen, R. A. Ophoff, S. A. Paciga, A. Palotie, C. N. Pato, S. Qin, M. Rietschel, B. P. Riley, M. Rivera, D. Rujescu, M. C. Saka, A. R. Sanders, S. G. Schwab, A. Serretti, P. C. Sham, Y. Shi, D. St Clair, H. Stefánsson, K. Stefansson, M. T. Tsuang, J. van Os, M. P. Vawter, D. R. Weinberger, T. Werge, D. B. Wildenauer, X. Yu, W. Yue, P. A. Holmans, A. J. Pocklington, P. Roussos, E. Vassos, M. Verhage, P. M. Visscher, J. Yang, D. Posthuma, O. A. Andreassen, K. S. Kendler, M. J. Owen, N. R. Wray, M. J. Daly, H. Huang, B. M. Neale, P. F. Sullivan, S. Ripke, J. T. R. Walters, M. C. O’Donovan, Schizophrenia Working Group of the

Psychiatric Genomics Consortium, Mapping genomic loci implicates genes and synaptic biology in schizophrenia. *Nature* **604**, 502–508 (2022).

84. C. A. de Leeuw, J. M. Mooij, T. Heskes, D. Posthuma, MAGMA: Generalized Gene-Set Analysis of GWAS Data. *PLoS Comput. Biol.* **11**, e1004219 (2015).

85. W. R. Reay, J. R. Atkins, Y. Quidé, V. J. Carr, M. J. Green, M. J. Cairns, Polygenic disruption of retinoid signalling in schizophrenia and a severe cognitive deficit subtype. *Mol. Psychiatry* **25**, 719–731 (2020).

86. W. R. Reay, M. J. Cairns, Pairwise common variant meta-analyses of schizophrenia with other psychiatric disorders reveals shared and distinct gene and gene-set associations. *Transl. Psychiatry* **10**, 134 (2020).

87. C. A. de Leeuw, B. M. Neale, T. Heskes, D. Posthuma, The statistical properties of gene-set analysis. *Nat. Rev. Genet.* **17**, 353–364 (2016).

88. M. J. Gandal, P. Zhang, E. Hadjimichael, R. L. Walker, C. Chen, S. Liu, H. Won, H. van Bakel, M. Varghese, Y. Wang, A. W. Shieh, J. Haney, S. Parhami, J. Belmont, M. Kim, P. Moran Losada, Z. Khan, J. Mleczko, Y. Xia, R. Dai, D. Wang, Y. T. Yang, M. Xu, K. Fish, P. R. Hof, J. Warrell, D. Fitzgerald, K. White, A. E. Jaffe, PsychENCODE Consortium, M. A. Peters, M. Gerstein, C. Liu, L. M. Iakoucheva, D. Pinto, D. H. Geschwind, Transcriptome-wide isoform-level dysregulation in ASD, schizophrenia, and bipolar disorder. *Science* **362**, eaat8127 (2018).

89. W. A. Banks, P. Sharma, K. M. Bullock, K. M. Hansen, N. Ludwig, T. L. Whiteside, Transport of extracellular vesicles across the blood-brain barrier: Brain pharmacokinetics and effects of inflammation. *Int. J. Mol. Sci.* **21**, 4407 (2020).

90. J. Kaur, L. M. Fahmy, E. Davoodi-Bojd, L. Zhang, G. Ding, J. Hu, Z. Zhang, M. Chopp, Q. Jiang, Waste clearance in the brain. *Front. Neuroanat.* **15**, 665803 (2021).
